# Antipurinergic Therapy Corrects the Autism-Like Features in the Poly(IC) Mouse Model

**DOI:** 10.1371/journal.pone.0057380

**Published:** 2013-03-13

**Authors:** Robert K. Naviaux, Zarazuela Zolkipli, Lin Wang, Tomohiro Nakayama, Jane C. Naviaux, Thuy P. Le, Michael A. Schuchbauer, Mihael Rogac, Qingbo Tang, Laura L. Dugan, Susan B. Powell

**Affiliations:** 1 The Mitochondrial and Metabolic Disease Center, University of California San Diego School of Medicine, San Diego, California, United States of America; 2 Department of Medicine, University of California San Diego School of Medicine, San Diego, California, United States of America; 3 Department of Pediatrics, University of California San Diego School of Medicine, San Diego, California, United States of America; 4 Department of Pathology, University of California San Diego School of Medicine, San Diego, California, United States of America; 5 Department of Neurosciences, University of California San Diego School of Medicine, San Diego, California, United States of America; 6 Department of Psychiatry, University of California San Diego School of Medicine, San Diego, California, United States of America; Torrey Pines Institute for Molecular Studies, United States of America

## Abstract

**Background:**

Autism spectrum disorders (ASDs) are caused by both genetic and environmental factors. Mitochondria act to connect genes and environment by regulating gene-encoded metabolic networks according to changes in the chemistry of the cell and its environment. Mitochondrial ATP and other metabolites are mitokines—signaling molecules made in mitochondria—that undergo regulated release from cells to communicate cellular health and danger to neighboring cells via purinergic signaling. The role of purinergic signaling has not yet been explored in autism spectrum disorders.

**Objectives and Methods:**

We used the maternal immune activation (MIA) mouse model of gestational poly(IC) exposure and treatment with the non-selective purinergic antagonist suramin to test the role of purinergic signaling in C57BL/6J mice.

**Results:**

We found that antipurinergic therapy (APT) corrected 16 multisystem abnormalities that defined the ASD-like phenotype in this model. These included correction of the core social deficits and sensorimotor coordination abnormalities, prevention of cerebellar Purkinje cell loss, correction of the ultrastructural synaptic dysmorphology, and correction of the hypothermia, metabolic, mitochondrial, P2Y2 and P2X7 purinergic receptor expression, and ERK1/2 and CAMKII signal transduction abnormalities.

**Conclusions:**

Hyperpurinergia is a fundamental and treatable feature of the multisystem abnormalities in the poly(IC) mouse model of autism spectrum disorders. Antipurinergic therapy provides a new tool for refining current concepts of pathogenesis in autism and related spectrum disorders, and represents a fresh path forward for new drug development.

## Introduction

Autism spectrum disorders (ASDs) are complex, multisystem disorders that are defined by unifying, core abnormalities in the development of language, social behavior, and repetitive behaviors. Hundreds of single-gene causes and chromosomal copy-number variations (CNVs) are known to confer risk, but in aggregate account for less than 20% of children with ASD [1]. More than 80% of children with ASD do not have a monogenic or CNV cause. The majority of children with ASD develop disease as the result of interactions between large sets of genes and environmental factors. Common comorbidities in non-single-gene forms of ASD provide important clues to shared mechanisms of disease. Comorbidities include epilepsy [2], GI abnormalities [3], sleep disturbances [2], abnormalities in tryptophan metabolism and platelet hyperserotonemia [4], altered intracellular calcium and mitochondrial dynamics [5], hypoimmunoglobulinemia [6], hyperuricosuria [7], methylation disturbances [8], disturbances in sulfur [9] and glutathione metabolism [10], neuroinflammation [11], cerebellar vermis hypoplasia [12], and Purkinje cell loss [13]. We hypothesized that all of these clinical comorbidities can result from a single, evolutionarily conserved, metabolic state associated with a cellular danger response (CDR). Since mitochondria are located at the hub of the wheel of metabolism and play a central role in non-infectious cellular stress [14], innate immunity [15], inflammasome activation [16], and the stereotyped antiviral response [17], we searched for a signaling system that was both traceable to mitochondria and critical for innate immunity. Purinergic signaling via extracellular nucleotides like ATP and ADP satisfied these requirements. In the following study we tested the role of purinergic signaling in the maternal immune activation mouse model of ASD and show that antipurinergic therapy reverses the abnormalities found in this model.

ATP, ADP, UTP, and UDP are mitokines—signaling molecules made by mitochondria—that act as signaling molecules when outside the cell, and have separate metabolic functions inside the cell. Outside the cell, they bind to and regulate purinergic receptors that are present on the surface of every cell in the body. ATP has been found to be a co-neurotransmitter at every type of synaptic junction studied to date [18]. Excess extracellular ATP (eATP) is an activator of innate and adaptive immunity [19], is a danger signal and damage-associated molecular pattern (DAMP) that is chemotactic for neutrophils [20], and a potent regulator of microglial activation, death, and survival [21]. The concentration of extracellular nucleotides under normal circumstances is ultimately controlled by mitochondrial function and cellular health.

Fifteen different isoforms of purinergic receptors are known that are stimulated by extracellular nucleotides [18]. These are divided into ionotropic P2X receptors and metabotropic P2Y receptors. P2Y receptors are G-protein coupled receptors (GPCRs). Together, P2X and P2Y receptors are known to control a broad range of biological characteristics that have relevance to autism. These include all the known abnormalities that occur in autism. For example, purinergic signaling modulates normal synaptogenesis and brain development [18], the PI3K/AKT pathway [22], innate and adaptive immune responses, and chronic inflammation [23], neuroinflammation, antiviral signaling [17], microglial activation, neutrophil chemotaxis, autophagy, gut motility [24], gut permeability [25], taste chemosensory transduction [26], sensitivity to food allergens [27], hearing [28], and chronic pain syndromes [18].

We hypothesized that the conserved cellular danger response (CDR) coordinates the metabolic responses to intracellular pathogens [17] and NRF2-coordinated electrophilic chemical stress [29]. In the MIA model of ASD, adult females are exposed to a simulated viral infection by injection of a synthetic, double strand RNA poly(Inosine∶Cytosine) (poly(IC)) at vulnerable times during pregnancy. This produces offspring with neurodevelopmental abnormalities associated with both ASD [30] and schizophrenia [31]. Injected poly(IC) RNA is not replicated, but is recognized by the antiviral response machinery within the cell. Poly(IC) binds to TLR3, the dsRNA-activated protein kinase (PKR), and other proteins, activates the cellular danger response, inhibits the translation of cap-dependent mRNAs, and stimulates IL1β, IL6, TNFα, and the type I interferons (IFNα and IFNβ). Poly(IC) exposure produces self-limited sickness behavior that is characterized by initial fever then hypothermia, decreased activity, reduced food and water intake, weight loss, and spontaneous recovery in about 24 hours [32].

We tested the hypothesis that the cell danger response (CDR) is sustained by hyperpurinergia. Suramin is a well-known and well-studied competitive inhibitor of purinergic signaling [33]. It has been used medically for the treatment of African Sleeping Sickness (Trypanosomiasis) since shortly after it was first synthesized in 1916. Its antipurinergic actions were discovered in 1988, after a search for inhibitors of ATP-mediated P2X and P2Y signaling [34]. We used suramin to test the role of purinergic signaling in the maternal immune activation (MIA) model of autism-like behaviors in C57BL/6J mice. In this study, we report for the first time the functional correction of both the core behavioral symptoms and multi-system comorbidities of the MIA model of autism spectrum disorders using a single drug that inhibits purinergic signaling. A total of 16 multisystem features of this model were either corrected or improved by suramin treatment (summarized in Table 1).

**Table 1:** Summary of Antipurinergic Therapy Results in the Poly(IC) Mouse Model of Autism Spectrum Disorders.

Feature	Abnormality in Males	Response to Antipurinergic Therapy
**Social Preference**	Decreased	Normalized (p<0.05)
**Sensorimotor Coordination (Rotarod)**	Decreased	Normalized (p<0.001)
**Basal Body Temperature**	Decreased	Normalized (p<0.001)
**Oxygen Consumption During Sleep**	Unchanged*	Increased (p<0.001)
**Plasma Immunoglobulins**	Unchanged*	Increased (p<0.05)
**Plasma Corticosterone**	Unchanged*	Increased (p<0.03)
**Synaptosomal Structure by Electron Microscopy**	Fragile and malformed post-synaptic densities; Accumulation of electron dense material	Normalized
**Cerebral Mitochondrial Respiratory Chain Complex I Activity**	Increased	Normalized (p<0.02)
**Cerebral Mitochondrial Respiratory Chain Complex IV Activity**	Increased	Normalized (p<0.02)
**Synaptosomal Purinergic Receptor (P2Y2) Expression**	Decreased	Normalized (p<0.02)
**Synaptosomal Purinergic Receptor (P2X7) Expression**	Decreased	Normalized (p<0.02)
**Synaptosomal ERK1/2 Phosphorylation**	Decreased	Normalized (p<0.001)
**Synaptosomal CAMKII Phosphorylation**	Decreased	Normalized (p<0.001)
**Synaptosomal FMRP Expression**	Decreased	Normalized (p<0.02)
**Synaptosomal Nictotinic Acetylcholine Receptor subunit α7 Expression**	Unchanged*	Increased (p<0.001)
**Cerebellar Vermis Lobule VII Purkinje Cell Number**	Decreased	Preserved (p<0.05)

* Unchanged at 4 months of age in the sham-treated poly(IC) mice, but increased therapeutically by suramin.

## Materials and Methods

### Animals and Husbandry

**Figure 1:**
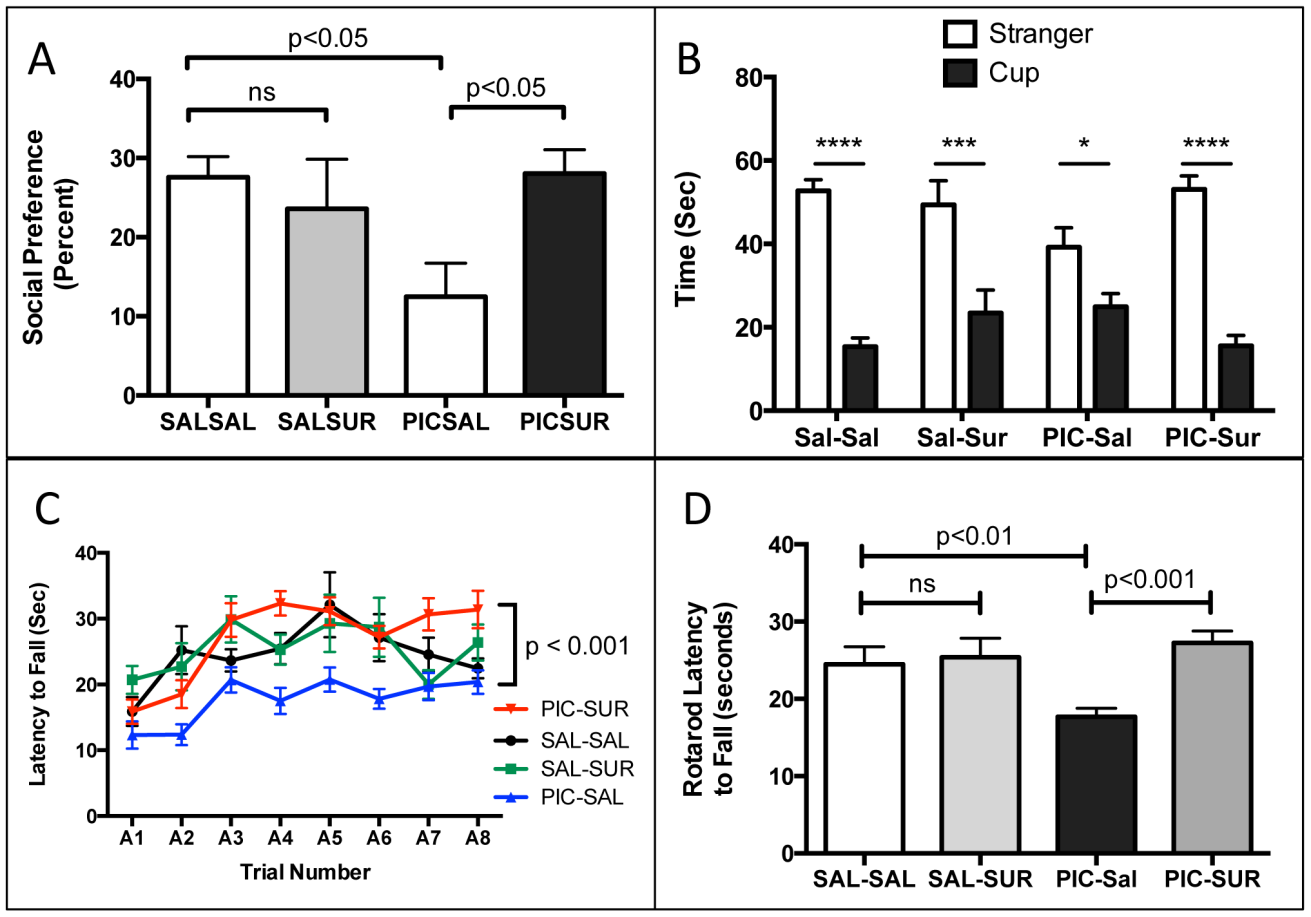
Correction of the Core Behavioral Features of the Maternal Immune Activation (MIA) Mouse Model of Autism Spectrum Disorders (ASD). (A) Social Preference. MIA males had a 54% decrease in social preference compared to controls (PIC-Sal 12.5+/−4.2% vs Sal-Sal 27.6+/−2.6%; one-way ANOVA F(3,42) = 3.74; with Newman-Keuls post-hoc testing; n = 9–15 males per group; age = 10-weeks; p<0.02). This was corrected by suramin treatment (PIC-Sur 28.1% vs Sal-Sal 27.6%; p = ns). (B) Social Preference as the time spent with stranger mouse vs. inanimate cup from 0–5 minutes. Analyzed by 2-Way ANOVA with Bonferroni pair-wise post testing (*p<0.05; ***p<0.001; ****p<0.0001). Treatment with suramin had little effect on normal behavior (Sal-Sal vs Sal-Sur), but a strong effect in improving social behavior in the MIA group (PIC-Sal vs. PIC-Sur). Zone x treatment interaction F(3,43) = 3.72; p<0.05; n = 9–15 males per group; age = 10-weeks. (C) Rotarod Training Curves. MIA (PIC-Sal) animals displayed deficits that were corrected by suramin treatment. Analyzed by repeated measures ANOVA with Tukey post testing: Sal-Sal vs. PIC-Sal q = 6.749, p<0.01; PIC-Sal vs PIC-Sur q = 11.13, p<0.001; n = 9–16 males per group; age = 11-weeks. (D) Rotarod Sensorimotor Coordination. MIA animals had a 28% decrease in sensorimotor coordination as measured by latency to fall by rotarod testing (PIC-Sal = 17.7+/−1.6 sec vs Sal-Sal = 24.5+/−2.2 sec; one-way ANOVA F(3,46) = 7.08; n = 9–16 males per group; age = 11-weeks; p<0.001). This was corrected by suramin treatment (PIC-Sur 27.2+/−1.6 sec vs Sal-Sal 24.5+/−2.2 sec; p = ns). Values are expressed as mean +/− SEM.

**Figure 2:**
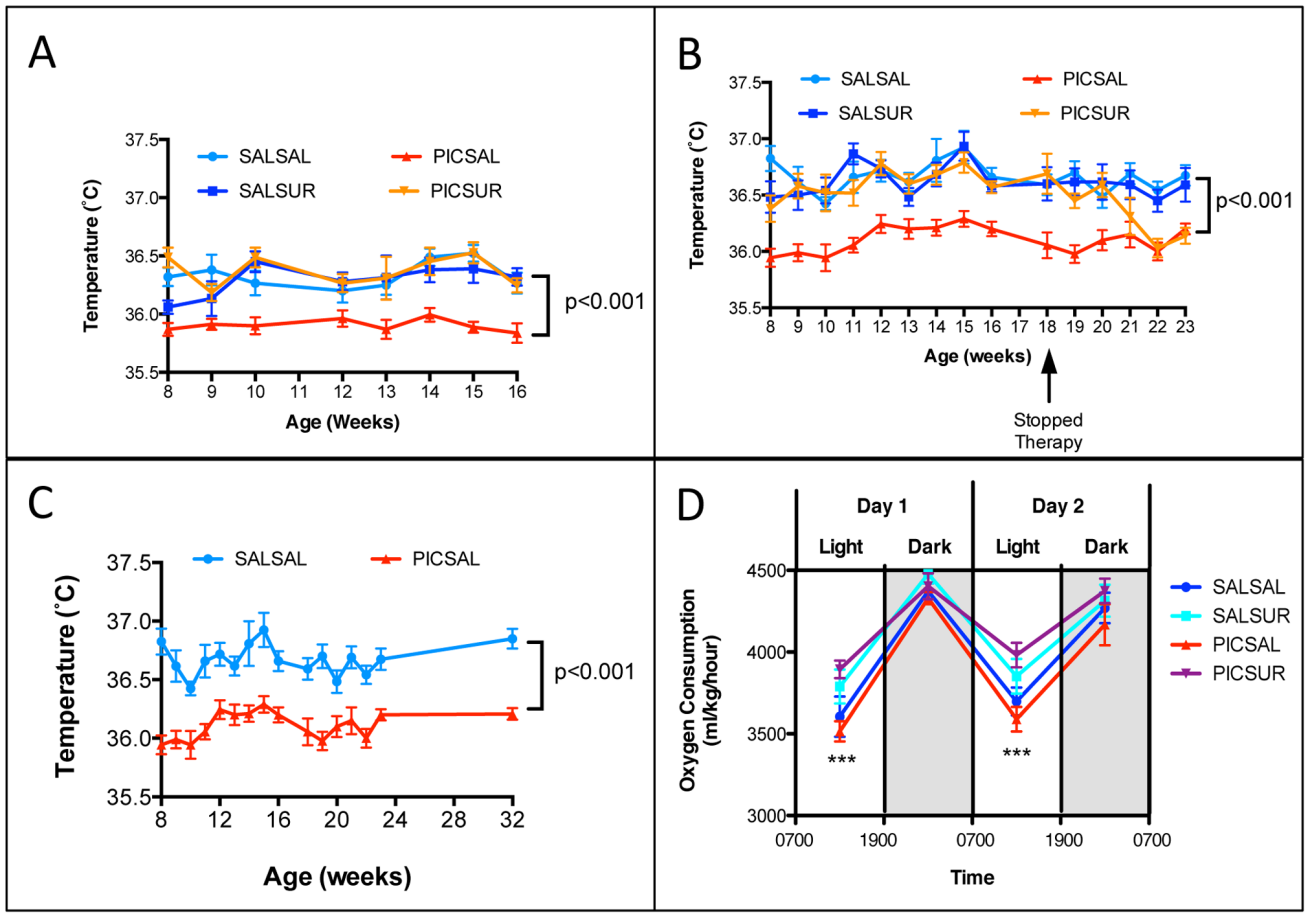
Relative Hypothermia Was Corrected, and Aerobic Metabolism was Increased by Antipurinergic Therapy. (A) Relative Hypothermia in the MIA Model was Corrected by Antipurinergic Therapy. (Linear mixed effects model analysis; F(1,47) = 25.3; n = 9–16 males per group; ages 8–16 weeks; p<0.001) (B) Correction of the Relative Hypothermia Was Lost After Discontinuing Antipurinergic Therapy. Weekly injections of suramin were discontinued in females at 18 weeks of age (PIC-SUR group; orange line, inverted triangles). By 22 weeks, hypothermia in the MIA animals returned to the untreated level approximately 0.5° below normal. (F(1,39) = 43.7; n = 9–16 females per group; p<0.001). (C) Relative Hypothermia is a Long-term Feature of the Poly(IC) MIA Model. Hypothermia persisted for at least 8 months of age (linear mixed effects model analysis F(1,19) = 114; n = 9–12 females per group; p<0.001). (D) Aerobic Metabolism. Oxygen consumption in the MIA animals showed a trend toward being decreased in both sleep (light) and active (dark) cycles. Suramin treatment increased sleep cycle oxygen consumption by 11%; MIA = PIC-Sal VO_2_ = 3552+/−47.6 ml/kg/hour; Treated MIA = PIC-Sur = 3938+/−45.9 (one-way ANOVA F(3,44) = 8.0; n = 6 males per group; age = 14 weeks; p = 0.0002). Antipurinergic therapy had no significant effect on oxygen consumption in the control animals; Saline-treated Controls = Sal-Sal VO_2_ = 3652+/−72.8; Treated Controls = Sal-Sur = 3821+/−71.5 (n = 6 males per group; p = 0.11). Values are expressed as mean +/− SEM.

**Figure 3:**
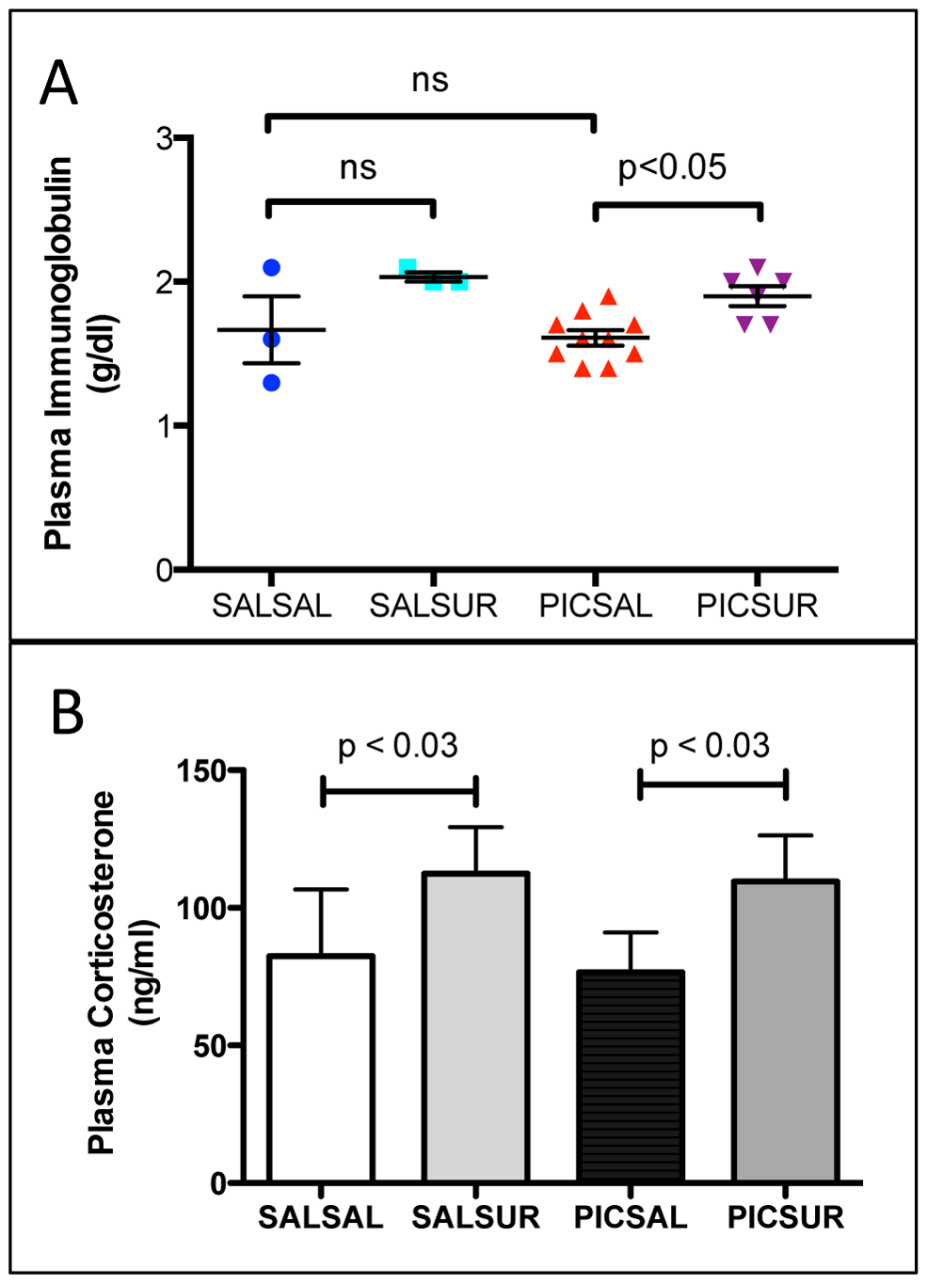
Plasma Immunoglobulins and Corticosterone. (A) Plasma immunoglobulins were increased 18% by antipurinergic therapy. (PIC-Sal = 1.6+/−0.05 mg/dl; PIC-Sur = 1.9+/−0.07 mg/dl; n = 6–10 males per group; age = 12 weeks; one-way ANOVA F(3,37) = 5.72; p<0.05) (B) Plasma corticosterone levels were increased 50% by weekly suramin treatment (PIC-Sal = 77+/−14 ng/ml; PIC-Sur = 117+/−16 ng/ml; two-way ANOVA F(1,37) = 5.16; p = 0.03; n = 8–12 males per group; age = 12 weeks). Values are expressed as mean +/− SEM.

**Figure 4:**
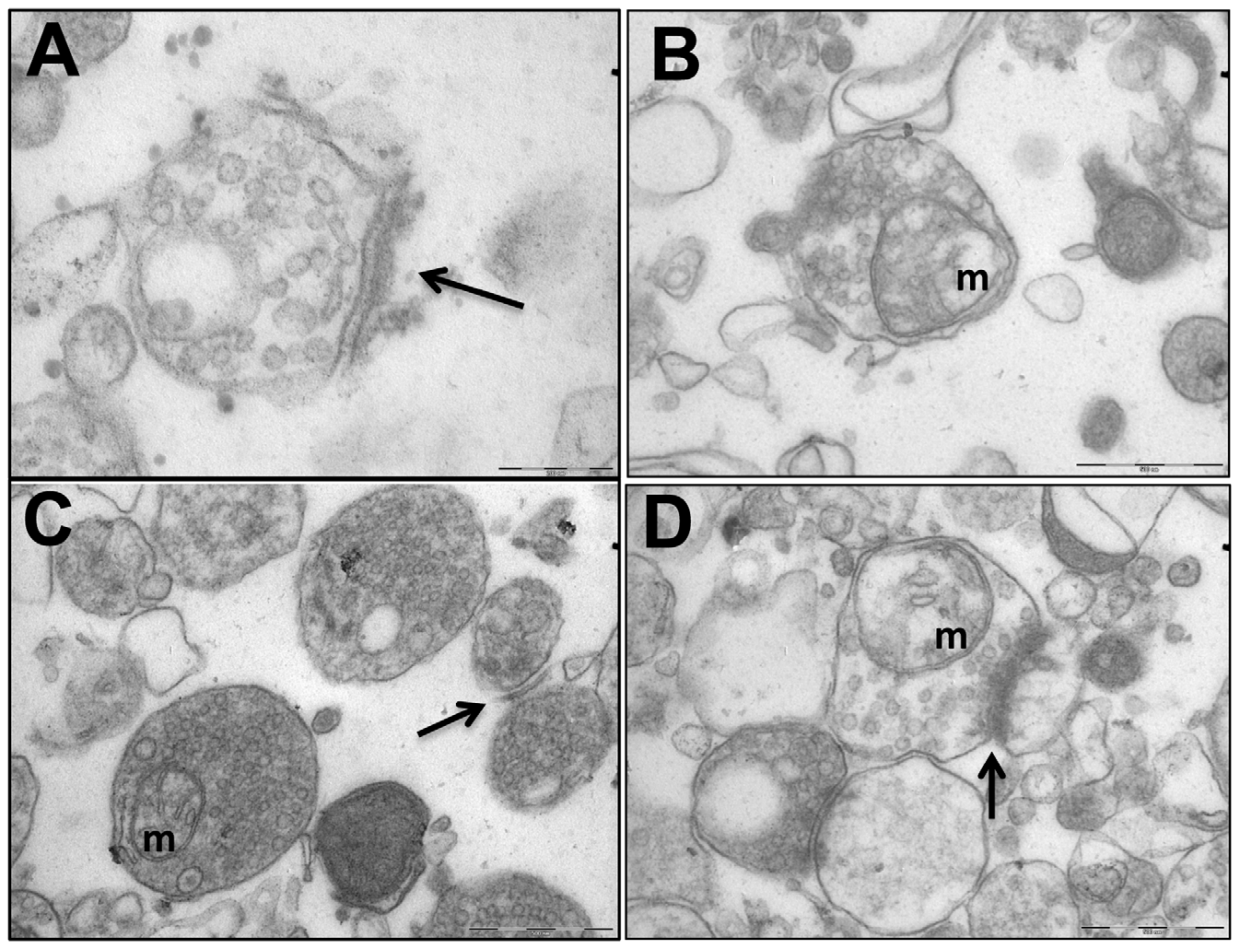
Cerebral Synaptosomal Ultrastructural Abnormalities Were Corrected by Antipurinergic Therapy. (A) Control (Sal-Sal) synaptosome illustrating normal post-synaptic density (PSD) morphology (arrow), and normal electron lucency of the matrix (92,000× magnification; scale bar = 200 µm). (B) Treated controls (Sal-Sur) with an included mitochondrion (“m”; scale bar = 500 µm). (C) Untreated MIA (PIC-Sal) with an included mitochondrion (“m”) and malformed, hypomorphic PSD (arrow; scale bar = 500 µm). Note the abnormal accumulation of electron-dense matrix material. (D) Treated MIA (PIC-Sur) with restoration of near-normal PSD morphology (arrow), an included mitochondrion (“m”), and reduction in abnormal accumulations of electron-dense matrix material within the synaptosomes (scale bar = 500 µm). Representative fields from n = 3–4 males per group; age = 16 weeks.

**Figure 5:**
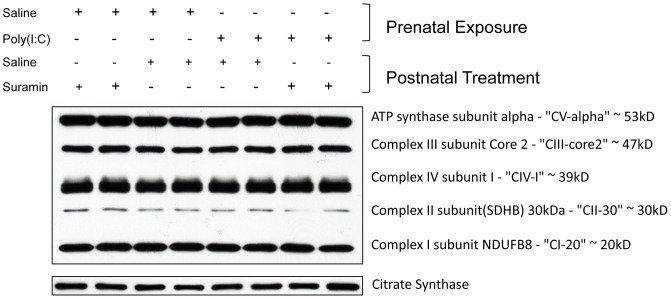
Cerebral Mitochondrial Respiratory Chain Subunit Mass was Unchanged in the MIA Model. Cerebral mitochondria were isolated by Percoll gradient centrifugation and quantified by Western Analysis. Each lane contains the mitochondria from 2–3 males isolated at 16-weeks of age (n = 4–5 per group).

**Figure 6:**
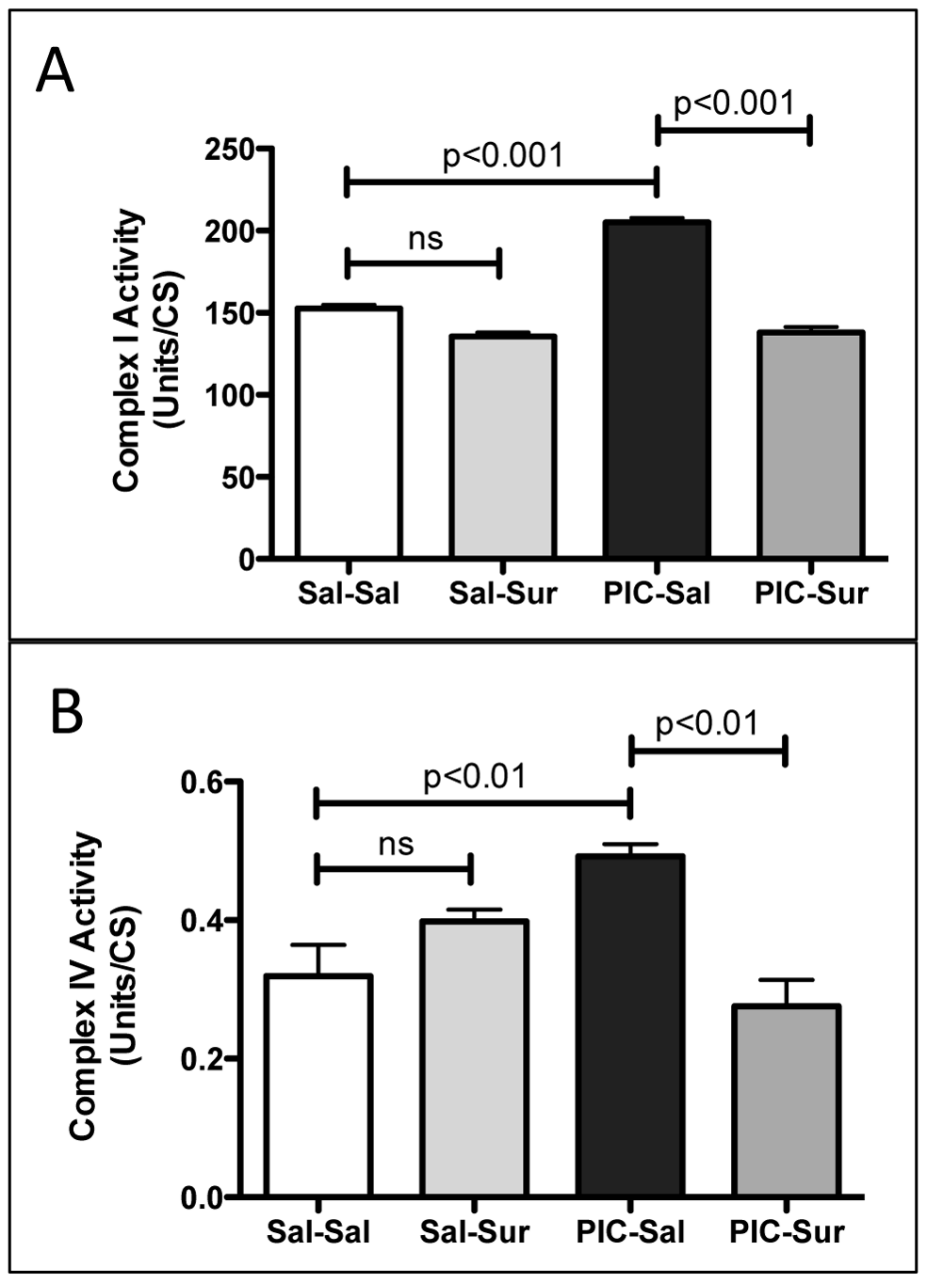
Cerebral Mitochondrial Respiratory Chain Hyperfunction Abnormalities Were Corrected by Antipurinergic Therapy. (A) Mitochondrial Respiratory Chain Complex I Enzymatic Activity was Increased 34% by gestational poly(IC) exposure and corrected by suramin treatment (Sal-Sal = 152+/−2.3 U/CS; PIC-Sal = 205+/−2.9 U/CS; one-way ANOVA F(3,12) = 137; p<0.0001; n = 4–5 males per group; age = 16 weeks). (B) Complex IV Activity was increased 53% by gestational poly(IC) exposure and corrected by suramin treatment (Sal-Sal = 0.319+/−0.045 U/CS; PIC-Sal = 0.492+/−0.018; one-way ANOVA F(3,12) = 8.9; p = 0.0022; n = 4–5 males per group; age = 16 weeks). Values are expressed as mean +/− SEM.

**Figure 7:**
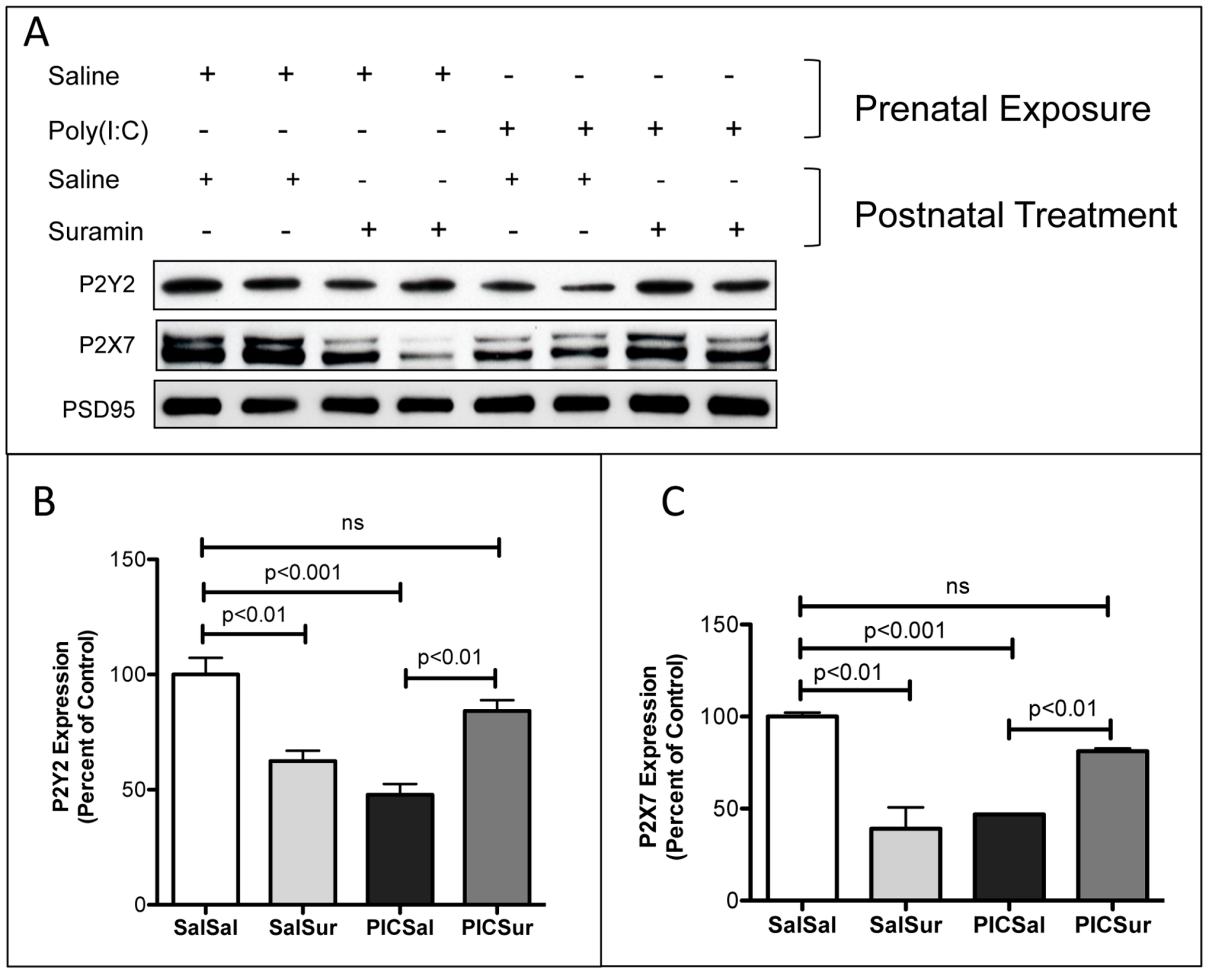
Cerebral Synaptosomal Purinergic Receptors were Downregulated in the MIA Model and Restored to Normal by Antipurinergic Therapy. (A) Western Analysis of Metabotropic P2Y2 and Ionotropic P2X7 receptors. Each lane contains the synaptosomes from 2–3 males isolated at 16-weeks of age (n = 4–5 per group). (B) P2Y2 receptor expression was decreased by over 50% by gestational poly(IC) exposure and normalized by suramin treatment (Sal-Sal = 100+/−7.3%; Sal-Sur = 62+/−4.6%; PIC-Sal = 48+/−4.7%; PIC-Sur = 84+/−4.7%; one-way ANOVA F(3,12) = 18.1; p<0.0001; n = 4–5 males per group). (C) P2X7 receptor expression was decreased over 50% by gestational poly(IC) exposure and normalized by suramin treatment (Sal-Sal = 100+/−2.2%; Sal-Sur = 39+/−12%; PIC-Sal = 47+/−0.5%; PIC-Sur = 81+/−1.5%; one-way ANOVA F(3,12) = 23.2; p<0.0001; n = 4–5 males per group). Post-synaptic density 95 (PSD95) protein was used as a loading control. Values are expressed as mean +/− SEM.

**Figure 8:**
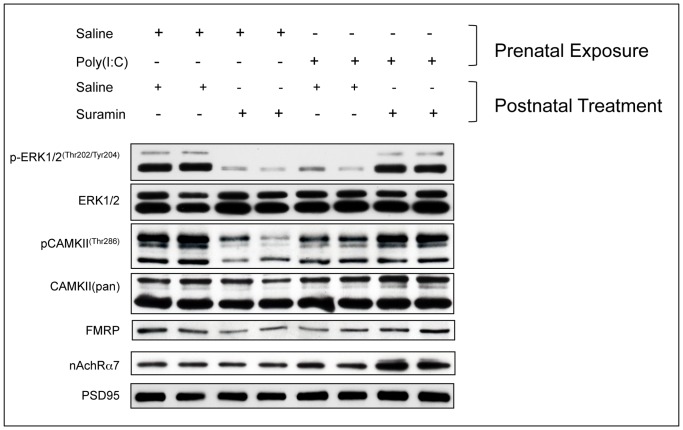
Cerebral Synaptosomal Hypophosphorylation of Extracellular Response Kinase 1 and 2 (ERK1/2), Calcium-Calmodulin Kinase II (CAMKII), and Downregulation Fragile X Protein (FMRP) in the MIA Model Were Corrected, and Nicotinic Acetylcholine Receptor subunit α7 (nAchRα7) Expression was Increased by Antipurinergic Therapy. (A) Western analysis of phosphorylated ERK1/2 (pERK1/2^Thr202/Tyr204^), total ERK1/2, phosphorylated CAMKII (pCAMKII^Thr286^), total CAMKII (CAMKIIpan), FMRP (Fragile X Syndrome protein), Nicotinic Acetylcholine Receptor subunit α7 (nAchRα7), and PSD95 (post-synaptic density protein 95) was used as a loading control for synaptosomes. Each lane contains the synaptosomes from 2–3 males isolated at 16-weeks of age (n = 4–5 per group).

**Figure 9:**
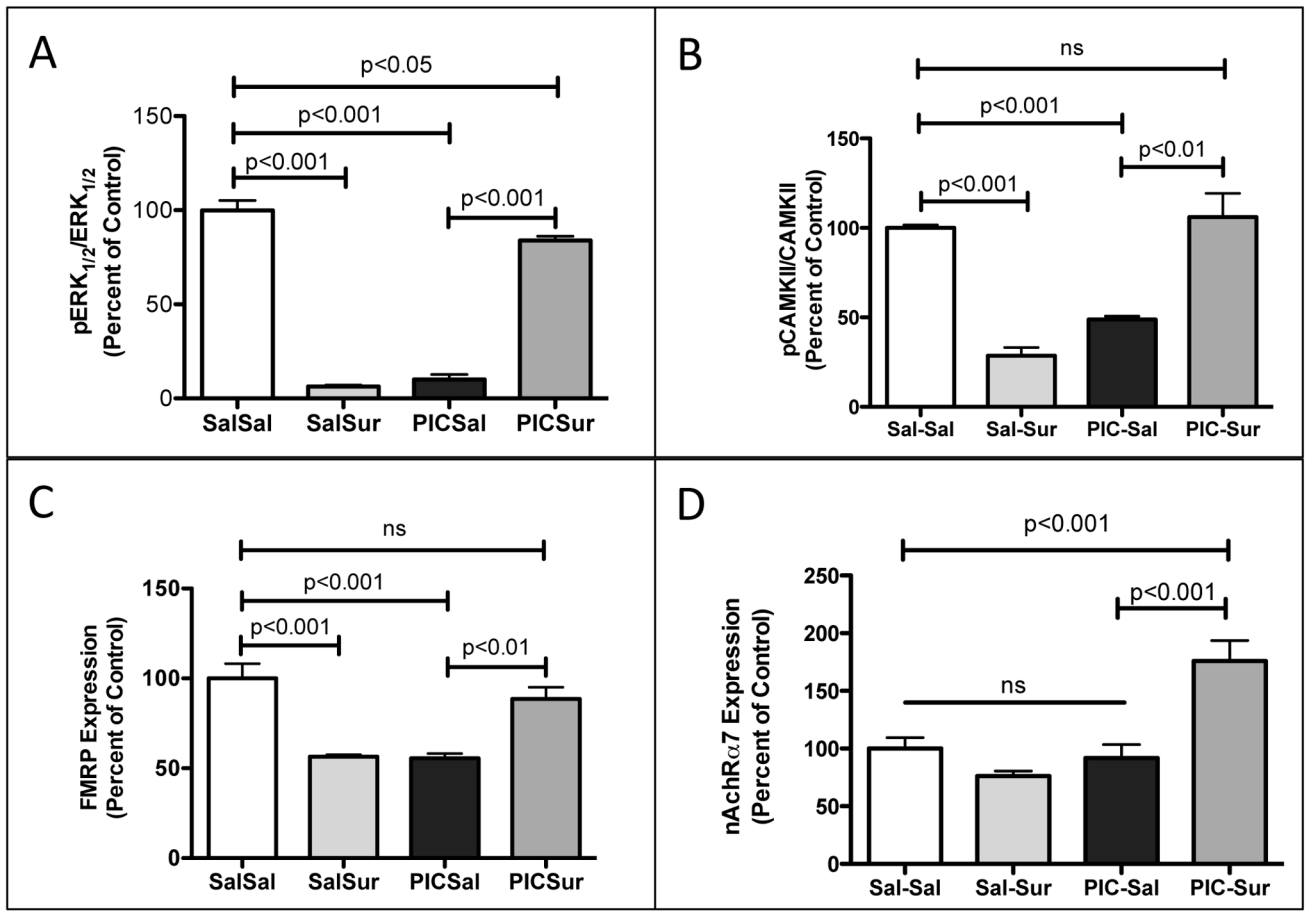
Quantitative Analysis of the Extracellular Response Kinase 1 and 2 (ERK1/2), Calcium-Calmodulin Kinase II (CAMKII), Fragile X Protein (FMRP), and the Nicotinic Acetylcholine Receptor subunit α7 (nAchRα7) in Cerebral Synaptosomes in the MIA Model. (A) ERK1 (MAPK3) and ERK2 (MAPK1) phosphorylation was decreased by over 90% by gestational poly(IC) exposure and normalized by suramin treatment (Sal-Sal = 100+/−5%; Sal-Sur = 6.2+/−0.8%; PIC-Sal = 9.9+/−2.6%; PIC-Sur = 84+/−2.3%; one-way ANOVA F(3,12) = 241; p<0.0001; n = 4–5 males per group; age = 16 weeks). (B) CAMKII phosphorylation was decreased by over 50% by gestational poly(IC) exposure and normalized by suramin treatment (Sal-Sal = 100+/−3%; Sal-Sur = 29+/−4.5%; PIC-Sal = 49+/−1.9%; PIC-Sur = 106+/−13%; one-way ANOVA F(3,12) = 25; p<0.0001; n = 4–5 males per group; age = 16 weeks). (C) Fragile X Mental Retardation Protein (FMRP) expression was decreased by over 40% by gestational poly(IC) exposure and normalized by suramin treatment (Sal-Sal = 100+/−8%; Sal-Sur = 56+/−1.2%; PIC-Sal = 55+/−2.5%; PIC-Sur = 89+/−6.5%; one-way ANOVA F(3,12) = 17.7; p<0.0001; n = 4–5 males per group; age = 16 weeks). (D) Nicotinic Acetylcholine Receptor subunit α7 (nAchRα7) expression was increased by over 75% by suramin treatment. (one-way ANOVA F(3,12) = 14.1; Sal-Sal = 100+/−9%; PIC-Sur = 176+/−18%; p<0.001; n = 4–5 males per group; age = 16 weeks; Newman-Keuls post test). Values are expressed as mean +/− SEM.

**Figure 10:**
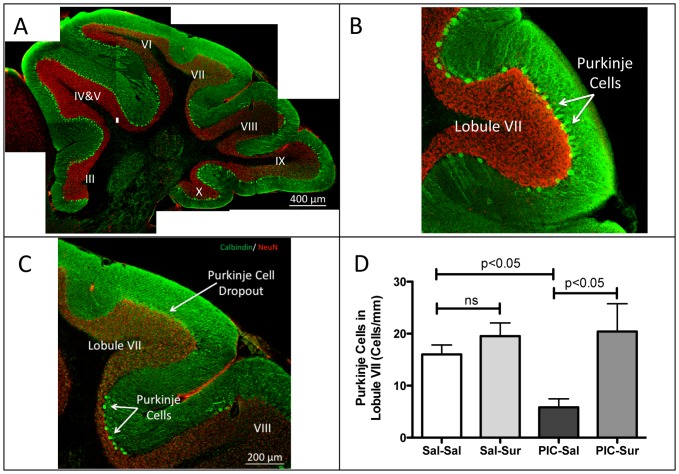
Purkinje Cell Dropout Was Prevented by Antipurinergic Therapy. (A) Mosaic reconstruction of a representative parasagittal section of the cerebellar vermis in an untreated MIA animal (PIC-Sal). Sections were stained for calbindin (green) and neuN (red). Purkinje cells are the bright, large neurons located at the margins of the molecular (green) and granular (red) layers of the cerebellum. Lobules III though X are indicated. MIA animals at 16 weeks of age, showed patchy loss of Purkinje cells that was most striking in lobules VI and VII. (B) Higher magnification of lobule VII in a control (Sal-Sal) animal illustrating normal Purkinje cell numbers. (C) Higher magnification of lobule VII in MIA illustrating nearly complete Purkinje cell dropout, with scattered cells at the boundary between lobules VII and VIII in an animal exposed to poly(IC) during gestation. (D) Quantitation of Lobule VII Purkinje Cells. Animals exposed to poly(IC) during gestation had a 63% reduction in Purkinje cell numbers. This was prevented by suramin treatment (10–20 mg/kg ip qWeek) started at 6 weeks of age (Sal-Sal = 16.0+/−1.8 cells/mm; Sal-Sur = 19.6+/−2.5; PIC-Sal = 5.8+/−1.6, PIC-Sur = 20.4+/−5.4; one-way ANOVA F(3,13) = 5.3; p = 0.013; Newman-Keuls post hoc test; n = 4–5 males per group; age = 16 weeks). Values are expressed as mean +/− SEM.

All studies were conducted at the University of California, San Diego (UCSD) in facilities accredited by the Association for Assessment and Accreditation of Laboratory Animal Care International (AAALAC) under UCSD Institutional Animal Care and Use Committee (IACUC)-approved animal subjects protocols. Six to 8-week old C57BL/6J (strain# 000664) mice were obtained from Jackson Laboratories (Bar Harbor, ME) and maintained on *ad libitum* Harlan Teklad 8604 mouse chow (14% fat, 54% carbohydrate, 32% protein) and water. Animals were housed in a temperature (22–24°C) and humidity (40–55%) controlled vivarium with a 12 h light-dark cycle (lights on at 7 AM). Two different protocols were used to produce the MIA model. In the first cohort, primiparous dams (experienced mothers) were mated at 12–14 weeks of age. Experienced sires were 4 months old. Seventy-five (75) offspring were studied from cohort 1. We used a higher-dose, two-dose protocol in the second cohort. In the second cohort, nulliparous dams were mated at 9–10 weeks of age and the sires were also 9–10 weeks of age. Ninety-three (93) offspring were studied from cohort 2. Behavioral and endocrine results from cohort 2 males are reported in Figures 1, 2, and 3. Brain biochemistry and synaptosome studies from cohort 1 males are reported in Figures 4, 5, 6, 7, 8, and 9. Brain cerebellar Purkinje cell results from cohort 2 males are reported in Figure 10. Temperature data from cohort 1 males and females appears in Table S1. Females generally displayed fewer and milder abnormalities than males in the poly(IC) MIA model (Figure S4). The results from long-term temperature measurements in females are reported in Figures 2B and 2C. Additional studies in both cohorts and both sexes are reported in the supporting online material (Tables S1, S2, and S3, and Figures S1, S2, S3, and S4). A total of 168 mice (86 males and 82 females) were studied.

### Poly(IC) Preparation and Gestational Exposure

To initiate the maternal immune activation (MIA) model pregnant dams received either a single intraperitoneal (ip) injection of Poly(I∶C) (Potassium salt; Sigma-Aldrich Cat# P9582; >99% pure; <1% mononucleotide content) of 0.17 A260 U/g; 2 mg/kg ip on E12.5 (cohort 1), or two doses (0.25 U/g [3 mg/kg] on E12.5 and 0.125 U/g [1.5 mg/kg] on E17.5) in cohort 2. This 2-dose poly(IC) regimen resulted in reduced fecundity of 40% (8 liveborn litters in 20 pregnancies; 95% CI = 19–64%) associated with fetal resorption after E12.5. Control animals injected with saline had a fecundity of 80% (8 liveborn litters in 10 pregnancies; 95% CI = 44–97%). There were no differences in liveborn litter size between saline and poly(IC)-injected pregnancies, which was 8.3+/−1.5 for 12–14 week-old primiparous dams, and 5.6+/−0.8 for 9–10 week-old nulliparous dams. P9582 vials contained nominally 50 mg of total solids (45 mg of PBS salts) and 5 mg of K-Poly(I∶C) lyophilized from 5 ml of PBS—hereafter referred to as a 50/5 vial. The contents of a 50/5 vial were reconstituted in 5 ml of sterile, nuclease-free water to yield an isotonic solution. Triplicate 5 µl samples of this solution were diluted 1∶200 in 1 ml of water and measured spectrophotometrically at 260 nm and 280 nm. A typical 50/5 vial contained 450 U of Poly(IC), and had an A260/A280 ratio of 1.65. The stock solution was then further diluted with a sufficient volume of 0.15M NaCl to produce an isotonic solution that was 50 A260 Units/ml. Using this concentration, a dose of 0.25 U/g is achieved using a volume of 5 µl/g, or 125 µl in a typical 25 g pregnant female.

### Postnatal Handling and Antipurinergic Therapy

Offspring of timed matings were weaned at 3–4 weeks of age into cages of 2–4 animals. No mice were housed in isolation. Littermates were identified by ear tags and distributed into different cages in order to minimize litter and dam effects. At 6-weeks of age, half the animals received a weekly injection of either saline (5 µl/g ip) or suramin (hexasodium salt, 10 or 20 mg/kg ip; Tocris Cat #1472). Beginning at 8-weeks of age all animals were evaluated by a series of test paradigms described below. At 16 weeks of age, male animals were sacrificed for synaptosome isolation, mitochondrial studies, hematology, blood chemistry, neuropathology, and immunohistochemical evaluation. Females from cohort 2 were followed for 8 months to monitor basal body temperatures and response to withdrawal of suramin treatment (Figs. 2B and 2C).

### Body Temperature Measurements

A BAT-12 Microprobe digital thermometer and RET-3 mouse rectal probe (Physitemp Instruments, Clifton, New Jersey) were used to obtain rectal core temperatures to a precision of +/−0.1°C. The probe was alcohol-cleaned, dipped in olive oil, then gently inserted 2 cm into the rectum for 10 seconds to achieve temperature and signal stability. Care was taken to avoid animal transport stress immediately prior to measurement in order to avoid stress-induced hyperthermia [35]. Temperatures were measured between 9 am to 12 noon each day.

### Locomotor Activity

Locomotor activity was tested in a brief open field test for 10 min in the light to assess anxiety-like behavior and subsequently in the mouse behavioral pattern monitor (mBPM) for 30 min in the dark to assess exploratory behavior and locomotor activity. Observers were blinded to treatment groups.

### Social Preference

Social preference was tested using a three-chambered box similar to what has been previously described [36]. Briefly, a Plexiglas box (60 cm L×60 cm W×30 cm H) was divided into 3 equal compartments by Plexiglas partitions containing an opening through which the mice could freely enter the 3 chambers. All testing was performed between the hours of 8 am and 1 pm. The test was conducted in two 10-minute phases. In phase I, the test mouse was first allowed to explore the chambers for 10 minutes. Each of the two outer chambers contained an empty, inverted stainless steel wire cup (Galaxy Cup, Spectrum Diversified Designs, Inc., Streetsboro, OH). In phase II, the test mouse was briefly removed, an unfamiliar mouse, age and sex matched, was placed under one of the wire cups and Lego blocks were placed under the other wire cup. The test mouse was then gently placed back in the arena and given an additional 10 minutes to explore. Room lighting for social behavior studies was 1–2 lux. An overhead camera and Ethovision v3 video tracking software (Noldus, Leesburg VA) were used to record the amount of time spent in each chamber and the number of entries into each chamber. In addition, a human observer, blinded to the treatment groups, scored time spent sniffing each wire cage, using a computer keypad. Stranger mice were used up to 4 times before new strangers were cycled in. The location (left or right) of the novel object and novel mouse alternated across subjects. Hand-scored times (seconds) with stranger and object were more sensitive than computer-calculated zone times (data not shown). Social preference (SP) in percent was calculated as 100 multiplied by the hand-scored time spent interacting with the stranger mouse (t_M_) divided by the sum of the time with stranger plus time with object (t_M_+t_L_) minus 50: SP = 100×[t_M_/(t_M_+t_L_)]−50. Total times spent interacting with stranger and Lego cup, as quantified by blinded observer, are presented in Figure 1B. Hand-scored and machine-scored results were similar (Figure S3).

### Rotarod

Training and testing were performed between the hours of 8 am and 1 pm using an accelerating rotarod protocol (Economex Rotarod, Columbus Instruments fitted with a 4 cm diameter spindle fitted with gray plastic, grooved walking surface) as previously described [37], with the following modifications. Prior to testing on an accelerating rod, mice were first trained at a fixed speed of 4 rpm. Each mouse was given up to 3 consecutive trials to achieve the endpoint of maintaining balance on the rotarod for at least 30 seconds. If a mouse was unsuccessful in the first 3 attempts, it was rested for 30 minutes, and then given another 3 attempts. Using this training protocol, all of the mice successfully maintained balance for 30 seconds within 2 training sessions. The acceleration phase testing was conducted over the subsequent 2 days, with 4 trials per day. Each mouse was individually placed on the rotarod at 4 rpm, which was then accelerated from 4 to 40 rpm over 5 minutes. The inter-trial time between repeat tests was 45 minutes. Latency to fall was recorded in seconds. Rotarod room lighting was 20–22 lux.

### Plasma Immunoglobulins, and Corticosterone

Whole blood (100–200 µl) was collected at 15 weeks of age, for immunoglobulin and corticosterone measurements by submandibular venous lancet (Goldenrod 5 mm) into heparinized or EDTA anticoagulated 0.3 ml Microtainer tubes (Becton-Dickinson). Blood draws were performed between the hours of 9 am and 1 pm to avoid known circadian variations in corticosterone and certain cytokines such as IL6. Plasma was separated by centrifugation at 1500 g×5 min and frozen at −80°C until use. Plasma immunoglobulins, albumin, and total protein were performed by the UCSD Animal Care Program Diagnostic Laboratory. Plasma concentrations of corticosterone were measured by ^125^I double antibody radioimmunoassay using 10 µl of plasma diluted 1∶200 with assay buffer (MP Biomedicals LLC, Orangeburg, NY). The intraassay coefficient of variation (CV) was 4% with an interassay CV of 7%.

### Comprehensive Metabolic Evaluations

Comprehensive Lab Animal Monitoring System (CLAMS, Columbus Instruments) cages were used to measure individual consumption rates of oxygen (VO_2_) and production rates of carbon dioxide (VCO_2_). The ratio of VCO_2_/VO_2_ is the Respiratory Exchange Ratio (RER). The RER was used to estimate the relative proportions of fat and carbohydrate utilized by each mouse provided the same diet of ad libitum Teklad 8604 mouse chow. The RER was then applied to the volume of gases exchanged to calculate energy expenditure in calories. In addition to gas measurements, feeding, drinking and total locomotor activity were also simultaneously measured. All of these measurements were made every 13 minutes for 48 hours starting after a 12-hour acclimatization period. Results were analyzed for each 12-hour interval of active (dark) and inactive/sleep (light) phases. Weights were measured prior to, and on completion of the experiment. Experimental data were exported from Oxymax (Windows) to Microsoft Excel and analyzed in GraphPad Prism.

### Synaptosome Isolation

Animals were sacrificed by cervical dislocation to prevent artifactual inhibition of mitochondrial function by all the known inhaled and injectable anesthetic agents. The brain was collected within 1 minute of sacrifice in 5 ml of ice cold BIOPS (K-MES 50 mM pH 7.1, Taurine 20 mM, Imidazole 20 mM, ATP 5.8 mM, MgCl_2_ 6.6 mM, Na_2_-Phosphocreatine 15 mM, DTT 0.5 mM K_2_-EGTA 10 mM, CaCO_3_ 2.8 mM; adjusted to pH 7.1). The wet weight was recorded to the nearest 0.1 mg. The right cerebri from two animals in the same treatment group were typically pooled and processed together. Nine volumes of BBG (0.32 M Sucrose, 1 mM K_2_-EDTA, 10 mM Tris pH 7.4, 10 mM glucose) were added and the brain was homogenized in a cold Glass-Glass Dounce (Kontes) homogenizer with 7–10 strokes. The homogenate was centrifuged at 3100 g×3 min at 4°C in a fixed angle SS34 rotor. The supernatant (S1) was collected and the pellet (P1) was homogenized again in 5 volumes of BBG. The homogenate was centrifuged at 1000 g×3 min. The supernatant (S2) was pooled with S1 and centrifuged at 16,000 g×10 min. The resulting pellet was resuspended in 4 ml of 15% Percoll in BB (0.32 M Sucrose, 1 mM K_2_-EDTA, 10 mM Tris pH 7.4). This suspension was carefully layered on a step gradient of 25% and 40% Percoll in BB (3.5 ml/each). The step gradient was centrifuged at 31,000 g×5 minutes at 4°C in a SS-34. Synaptosomes in band #2 at the 15%/25% Percoll interface were diluted with 10 volumes BB and centrifuged at 16,000 g×10 min. The pellet contained the synaptosomes. The synaptosomal pellet was resuspended in 5 volumes of SB (120 mM NaCl, 4.7 mM KCl, 2.2 mM CaCl_2_, 1.2 mM MgCl_2_, 25 mM HEPES, 1.2 mM MgSO_4_, 1.2 mM KH_2_PO_4_, 10 mM glucose) and centrifuged at 16,000 g×10 min. This removed sucrose and EDTA, which can interfere with many mitochondrial assays. The washed synaptosomal pellet was resuspended in 2 tissue volumes of SB.

### Synaptosome Electron Microscopy

Cerebral synaptosomes were drop dialyzed against water for 15 minutes, and 100 µg was pelleted by centrifugation at 16,000 g×10 minutes. Pellets were fixed in 3% glutaraldehyde in 0.1M cacodylate buffer, and after a brief wash, post fixed in 1% osmium tetroxide and subsequently dehydrated in graded ethanol series, treated in propylene oxide and embedded in EMbed 812/Araldite (Electron Microscopy Sciences, Hatfield PA). Thick sections (2 µm) were cut, mounted on glass slides and stained in toluidine blue for general assessment in the light microscope. Subsequently, 70 nm thin sections along the centrifugal gradient (from top to bottom of the pellet to assess for sedimentational sorting) were mounted on copper slot grids coated with parlodion and stained with uranyl acetate and lead citrate for examination at 80 kV on a Philips CM100 electron microscope (FEI, Hillsbrough OR). Images in tif format were documented using a Megaview III CCD camera (Olympus Soft Imaging Solutions, Lakewood CO).

### Brain Neuropathology and Confocal Microscopy

Brains were removed after 4% paraformaldehyde (PFA) perfusion-fixation of 4–5 randomly selected animals per group, and post-fixed for 6–24 hours in 4% PFA in PBS. Para-sagittal sections of the cerebellar vermis (50 µm) were cut with a Vibratome (The Vibrotome Company, St. Louis). Floating sections were blocked in PBS containing 5% horse serum, 1% BSA, and 0.3% Triton X-100 for 1 hour at room temperature, and incubated overnight at 4°C with antibodies against calbindin (Swant, PO Box 327, CH-1723 Marly, Switzland CB-38a, 1∶3000), and NeuN (Millipore,Temecula, MAB377, 1∶1000). Sections were then washed and stained with secondary antibodies for 2 hour at room temperature (Alex488-conjugated antibody against rabbit and Alex568-conjugated antibody against mouse, Invitrogen). Immunostained slices were mounted on coverslips, and imaged on a dedicated Zeiss LSM510 confocal imaging system. Cerebellar Purkinje cells were identified by calbindin staining in green at the interface of the molecular and granular layers of the cerebellar folia. Granular layer neurons were identified by NeuN staining in red.

### Western Blot Analysis

Ten µg of cerebral synaptosomal, or 2 µg of isolated mitochondrial protein was loaded in SDS-polyacrylamide gels (Bis-Tris Gels) and transferred to PVDF membranes. Blots were probed with primary antibodies overnight in cold room using anti-P2Y2 (#APR-010) and anti-P2X7 (#APR-004) antibodies from Alomone Labs (Jerusalem, Israel), anti-ERK1/2 (#4695), anti-phospho-Erk1/2 (Thr202/Tyr204) (#4370), anti-CAMKII (pan) (#4436), anti-phospho-CaMKII-Thr286 (#3361), anti-PSD95 (#3450) and anti-FMRP (#4317) antibodies from Cell Signaling (Danvers, MA, U.S.A.). Mitochondrial total OXPHOS antibody cocktail (#MS604) antibodies were purchased from MitoSciences (Eugene, Oregon, U.S.A.), anti-Citrate Synthetase (CS) (#ab96600) and anti-Nicotinic Acetylcholine Receptor α7 subunit (nAchRα7) (#ab23832) antibodies were purchased from Abcam (Cambridge, MA). After washing, the membranes were blotted with 1∶5000 diluted second antibodies in 5% milk/PBST for 1 hour at room temperature (goat anti-rabbit (#31460) and anti-mouse (#31430) second antibodies from Pierce (Rockford, IL USA). The proteins of interest were visualized by ECL reagent (Pierce, Cat#32109) or Pierce SuperSignalTM West Femto Maximum Sensitivity Substrate (Cat #PI-34095) and the immunoblots were exposed to X-Omat Blue films (Kodak) and scanned (Epson Perfection 2450 scanner). Bands were quantified using ImageJ 1.43u software.

### Respiratory Chain Enzymology

The enzymatic activity of mitochondrial complex I was measured as NADH:CoQ_1_ oxidoreductase activity by the method of Hatefi [38]. Complex II was measured as succinate:CoQ_1_ oxidoreductase activity by the method of Barrientos [39]. Complex II/III was measured as succinate: cytochrome c reductase activity by the method of Stumpf and Parks [40]. Complex III was measured as decyl-CoQ:cytochrome c reductase activity by the method of Barrientos [39] and expressed as a first order rate constant. Complex IV was measured as cytochrome c oxidase activity by the method of Wharton and Tzagoloff [41] and expressed as an apparent first order rate constant. Citrate synthase activity was used as a marker of mitochondrial mass and was measured by the method of Shepherd and Garland [42]. Rates were expressed as the ratio of respiratory chain enzyme activity to citrate synthase activity.

### Statistical analysis

Animals were randomized into active (suramin) and mock (saline) treatment groups upon weaning. Group means and standard error of the means (SEM) are reported. Data were analyzed using one-way ANOVA with treatment group as a between subject factor. One-way ANOVAs were used to test combined drug treatment and prenatal exposure effects on oxygen consumption and CO_2_ production (GraphPad Prism 5.0 d). Specific post hoc comparisons between selected groups were done using Newman-Keuls method. Body temperatures were analyzed using a linear mixed effects model with time as a within subject factor (SPSS Version 20). Significance was set at *p*<0.05 and indicated numerically.

## Results

### Social Behavior

Male offspring exposed to poly(IC) *in utero* showed 54% reduction in social preference. This was corrected by antipurinergic therapy (Fig. 1A). Social deficits in the MIA females were milder and more variable than males in the two cohorts studied (Figure S4A). We focused on male ASD-like phenotypes in the remainder of this study. There were no effects of poly(IC) or suramin treatment on locomotor activity (data not shown).

### Sensorimotor Coordination Deficits

Males also showed a 28% decrease in sensorimotor coordination as measured by latency to fall on rotarod testing. This was corrected by antipurinergic therapy (Fig. 1B). Female offspring born after the 2-dose poly(IC) protocol did not show significant rotarod abnormalities (Figure S4C).

### Relative Hypothermia

Both male and female MIA animals showed relative hypothermia of about 0.5°C below the basal body temperature of controls that persisted for the life of the animals (Figs. 2A–C). The magnitude of this effect was similar in both males and females (Fig. 2A, 2B), and both cohorts (Tables S1 and S2). Normal basal body temperature was restored by antipurinergic therapy within as little as two weeks of starting therapy at 6 weeks of age in both males (Fig. 2A) and females (Fig. 2B; Tables S1 and S2). Antipurinergic therapy had no effect on the body temperature of control animals (Sal-Sur). When antipurinergic therapy was stopped at 18 weeks of age, MIA (PIC-Sur) animals reverted to their previous level of relative hypothermia (36.1°C+/−0.1°) within 1 month, while control animals maintained normothermia (36.6°C+/−0.1°) (Fig. 2B). Hypothermia resulting from gestational exposure to poly(IC) appears to be permanent unless treated with a purinergic antagonist. It has lasted for at least 8 months—the age of our oldest animals available for study (Fig. 2C).

### Aerobic Metabolism

During the 12-hour period of light (7 am to 7 pm), during which the animals sleep, antipurinergic therapy increased the oxygen consumption of the MIA animals by 11% (Fig. 2D; Table S2). Antipurinergic therapy had no significant effect on oxygen consumption in the control animals (Fig. 2D). The CO_2_ production rates were proportionately increased so that the respiratory exchange ratios were unchanged between groups within sleep and active cycles (Table S2). There were no differences between groups in body mass index (BMI = mass in grams ÷ anal-snout distance in cm^2^), locomotor activity, weight gain, food, or water consumption during either light or dark phases. These results support the notion that antipurinergic therapy selectively increased aerobic (mitochondrial) metabolism and basal body temperature in MIA animals, and that these effects were greatest during sleep.

### Plasma Immunoglobulins and Corticosterone

We measured plasma immunoglobulins because these are reduced in children with autism, and increased levels correlate with decreased symptom severity [6]. Plasma immunoglobulins were not different between control and MIA animals, although our statistical power was limited by having only 3 control (Sal-Sal) animals available for blood chemistries in this experiment. On the other hand, antipurinergic therapy increased plasma immunoglobulins by about 20% (Fig. 3A) and the ratio of globulin to total protein (Figure S1).

We measured plasma corticosterone levels because high-dose suramin (up to 200 mg/kg) used in cancer clinical trials can produce adrenocortical insufficiency [43]. Plasma corticosterone levels were increased in males by about 50% by the weekly, low-dose (10–20 mg/kg ip) suramin treatment used in our study (Fig. 3B). Basal plasma corticosterone levels were 2–3 fold higher in females (Figure S2A), but were not changed by suramin treatment (Figure S2B).

### Synaptosomal Ultrastructural Abnormalities

Transmission electron microscopy of cerebral synaptosomes revealed significant differences between groups (Fig. 4). Control animals exhibited normally formed post-synaptic densities (PSDs) (arrow; Fig. 4A) and an electron-lucent synaptosomal matrix (Fig. 4A). Control animals receiving antipurinergic therapy were qualitatively similar to saline-treated controls (Fig. 4B). Striking differences were observed in the synaptosomes of the MIA animals. The large majority of synaptosomes contained an unidentified electron-dense matrix material (Fig. 4C) and the post-synaptic densities were fragile (easily disrupted during preparation), malformed, or both (arrow; Fig. 4C). Antipurinergic therapy of the MIA animals decreased the electron dense matrix material and restored more normal PSD architecture (arrow; Fig. 4D).

### Cerebral Mitochondrial Respiratory Chain Biochemistry

Respiratory chain complexes I, III, and IV assemble to form a supercomplex in the brain and other tissues [44]. We purified cerebral mitochondria and found no change in the protein mass of the core subunits of complexes I, II, III, IV, and V measured by immunoblot analysis, or of the mass of the mitochondrial matrix marker citrate synthase (Fig. 5). In contrast, we found a 34% increase in the enzymatic activity of respiratory chain Complex I activity (NADH:CoQ1 oxidoreductase) (Fig. 6A) and a 53% increase in Complex IV activity (Cytochrome c Oxidase) (Fig. 6B). These mitochondrial respiratory chain hyperactivity abnormalities were corrected by antipurinergic therapy (Fig. 6A, 6B).

### Synaptosomal Purinergic Receptors

Testing the hypothesis that purinergic signaling is chronically increased in the MIA model of ASD cannot be achieved by measuring tissue or plasma concentrations of nucleotides like ATP and ADP. The relevant concentration of nucleotides is confined to a thin shell, or pericellular halo, that defines the unstirred water layer (UWL) around the effector cells where receptors and their ligands meet. Concentrations of metabolites in the UWL can be 1000-fold higher than in plasma or interstitial fluid [45]. Hence, we selected purinergic receptor downregulation as a surrogate for chronic hyperpurinergia. Immunoblot analysis of cerebral synaptosomes showed 50–60% reduction in the expression of P2Y2, and P2X7 receptors in the MIA animals. These abnormalities were corrected by antipurinergic therapy (Fig. 7A–C).

We also noted downregulation of purinergic receptors in the synaptosomes of non-MIA control animals treated with suramin (SalSur; Fig. 7A–C). However, downregulation by chronic inhibition of purinergic signaling by suramin treatment alone did not produce any behavioral abnormalities in these control animals. The finding that antipurinergic therapy had opposite biochemical effects in healthy and ASD-like animals, and no behavioral effects in healthy animals, emphasizes the importance of more distal steps in the purinergic signaling cascade that are not addressed in this study. P2Y6 and the P1 adenosine receptors (A1, A2A, A2B, and A3) were not expressed at levels detectable by femto-ECL in cerebral synaptosomes (data not shown).

### Synaptosomal ERK1/2 and CAMKII Signaling

We next quantified ERK1 and 2 and CAMKII phosphorylation because they are known effectors of P2Y2- and P2X7-mediated purinergic signaling [22], [46]. We found a 90% reduction in the phosphorylation of ERK1 (MAPK3) and 2 (MAPK1), and a 50% reduction in the phosphorylation of calcium/calmodulin-dependent protein kinase II (CAMKII) (Fig. 8, 9A, 9B). These abnormalities were corrected by antipurinergic therapy. Treatment of non-MIA control animals with suramin also resulted in hypophosphorylation (SalSur; Fig. 8, 9A, 9B). This effect is opposite of the effect of suramin in MIA (PIC-Sur) animals. No behavioral changes or toxicities were observed in the control animals treated with suramin. This suggests that both the behavioral and biochemical responses to antipurinergic therapy were dependent on the physiologic state of the animal being treated.

### Synaptosomal FMRP Deficits

We next tested our hypothesis that chronic innate immune activation by hyperpurinergia would result in the downregulation of the Fragile X Mental Retardation Protein (FMRP) to facilitate inflammatory cytokine expression. This occurs because FMRP inhibits the translation of many inflammatory cytokines through AU-rich elements (AREs) in the 3′-untranslated regions of their respective mRNAs [47], and must be downregulated to permit increased cytokine translation. We found that FMRP expression was decreased by nearly 50% in the MIA males and restored to normal with antipurinergic therapy (Fig. 8, 9C). Treatment of non-MIA control animals also decreased synaptosomal FMRP expression (SalSur; Fig. 8, 9C).

### Synaptosomal nAchRα7 Expression

We tested the expression of the nicotinic acetylcholine receptor subunit α7 (nAchRα7) because of its role as an anti-inflammatory regulator of innate immunity [48] and its promise as a therapeutic target in schizophrenia and other disorders [49]. We found that nAchRα7 expression was not changed in untreated MIA animals (PIC-Sal; Fig. 8, 9D). However, suramin treatment of these animals increased the expression of this cholinergic receptor by over 75% (PIC-Sur; Fig. 9D).

### Cerebellar Purkinje Cell Dropout

Some of the first structural brain abnormalities to be reported in autism were examples of volume loss in the brainstem and cerebellar vermis that was most significant in lobules VI and VII [12], [50]. We wished to quantify cerebellar Purkinje cells in the MIA model because Purkinje cell dropout is a characteristic feature of certain primary mitochondrial disorders such as Alpers syndrome [51] and because Purkinje cell dropout in lobule VII of the cerebellar vermis is also a feature of decreased FMRP expression in Fragile X Syndrome [52] and in the MIA mouse model [53]. We found evidence of patchy Purkinje cell loss (Fig. 10A) that was marked in Lobule VII (Fig. 10B–D). Quantitative analysis showed a 63% loss of Purkinje cells in the MIA (PIC-Sal) ASD animals by 16-weeks of age (PIC-Sal; Fig. 10D). Antipurinergic therapy, starting at 6-weeks of age, prevented Purkinje cell loss measured at 4 months of age (PIC-Sur; Fig. 10D). This is consistent with the hypothesis that Purkinje cell survival and loss are occurring dynamically throughout the first few months of life in the MIA mouse model and that suramin treatment slows the rate of Purkinje cell loss.

## Discussion

The purpose of our study was to test the role of purinergic signaling abnormalities in a mouse model of ASD, and to test a new approach to treatment that targeted these abnormalities. We did not start treatment until 6-weeks of age, near the onset of reproductive maturity in the mouse, because we wished to test the hypothesis that many of the autism-like features of the MIA model were treatable after they appear, and are not fixed. No animal model is a perfect surrogate for human autism. However, the maternal immune activation (MIA) model, using poly(IC) exposure to simulate a viral infection during pregnancy, has been used extensively over the past decade to study the detailed neurodevelopmental abnormalities associated with both ASD [30] and schizophrenia [31]. Maternal fever in humans is a known risk factor for ASD [54]. This mouse model can be adjusted in severity and character according to the dose of poly(IC) used, and the timing of exposure during pregnancy. In this report we used either one or two gestational exposures to poly(IC). The one-exposure paradigm of poly(IC) given on E12.5 produced biochemical and metabolic abnormalities, but weaker behavioral and sensorimotor coordination abnormalities. The two-exposure paradigm on E12.5 and E17.5 magnified these effects and permitted more in-depth analysis of the ASD-like features of the MIA model. We found that all the abnormalities that were produced by poly(IC) were corrected by treatment with suramin (Table 1).

Perhaps our most striking observation was the preservation of cerebellar Purkinje cells in lobule VII with antipurinergic therapy (Fig. 10). It has been shown that Purkinje cell loss is a consistent feature of the MIA mouse model of autism [53]. This is especially prominent in lobules VI and VII of the cerebellar vermis [53], and represents a strong point of shared biology between human ASD and the MIA model. One of the first structural brain abnormalities found in children with ASD was hypoplasia of the cerebellar vermis that preferentially affected lobules VI and VII [12]. This has also been documented in adults with Fragile X Syndrome [52]. Cerebellar Purkinje cells are large, fast-spiking (ca. 50 Hz), GABAergic, inhibitory neurons that are particularly sensitive to bioenergetic supply and demand problems, and to toxic exposures [55]. Our finding of preserved cerebellar Purkinje cell numbers at 16 weeks of age in the MIA model with antipurinergic therapy supports the notion that the rate of postnatal Purkinje cell loss is dynamic and can be regulated by environmental factors. In the MIA mouse model, antipurinergic therapy slows the rate of Purkinje cell loss from 6 to 16 weeks of age.

Like human autism spectrum disorders, the MIA mouse model of ASD has both core behavioral abnormalities, and multisystem comorbidities that emerge as a consequence of underlying metabolic disturbances. Our results support the paradigm that all of the observed metabolic disturbances in this model are a manifestation of the conserved cell danger response (CDR). The CDR therefore lies at or near the root cause of the neurodevelopmental and biochemical abnormalities that characterize the ASD-like features in this model. Extracellular ATP is a mitokine and well-known danger signal [19] that we hypothesized initiates and sustains the cellular danger response in autism spectrum disorders. In related studies we found that direct systemic injection of nucleotides like ATP and ADP caused rapid hypothermia by decreasing mitochondrial oxygen consumption and tissue oxygen demand (VO_2_, data not shown). Hypothermia from systemic nucleotide injection has been studied in the fields of torpor and hibernation physiology [56]. We found that a convenient marker of the persistent cellular danger response in the poly(IC) model is relative hypothermia of about 0.5°C. Hypothermia was associated with an increase in the maximal enzymatic rates, but not the mass, of brain mitochondrial respiratory chain complexes I and IV. Treatment with suramin decreased brain mitochondrial activity to normal, increased the whole body oxygen consumption (metabolic rate, VO_2_) in the MIA animals, and increased the body temperature to normal (Fig. 2). The combination of higher mitochondrial electron transport activities measured *in vitro* and decreased basal oxygen consumption measured *in vivo* implies a novel increase in mitochondrial coupling efficiency and increased reserve capacity in ASD that is similar to that seen with exercise training [57]. We did not further investigate this phenomenon in this study.

Purinergic P2Y2 receptors and their phosphorylated effectors, ERK1/2 and CAMKII, are downregulated by chronic nucleotide stimulation in a process that leads to desensitization [58]. Our finding of downregulation of these purinergic receptors and their effectors is strong evidence for chronically elevated purinergic signaling in the poly(IC) model. Together, these findings are consistent with the notion that hyperpurinergia is a causal factor that initiates and maintains the cellular danger response in the MIA model of ASD. Suramin treatment corrected both the hyperpurinergia and the multisystem abnormalities in this model (Table 1).

We quantified the expression of the Fragile X protein (FMRP) in cerebral synaptosomes because deficiency is a cause of autism spectrum disorders, and normal expression inhibits the translation of several cytokines induced by innate immune activation [47]. Since innate immunity is persistently activated in the MIA model, we expected to find FMRP to be downregulated. We found that synaptosomal FMRP was decreased by about 50% in the MIA model and that antipurinergic therapy restored normal levels (Fig. 9C). This supports the notion that FMRP is downregulated as part of the multi-system abnormalities found in the MIA model even though the animals are not genetically deficient in the Fragile X (*FMR1*) gene. These observations are consistent with the hypothesis that FMRP down-regulation is part of the generalized cellular danger response produced by hyperpurinergia in this model of autism spectrum disorders.

Suramin treatment strongly increased the expression of the nicotinic acetylcholine receptor subunit α7 (nAchRα7) in cerebral synaptosomes of MIA animals, but had no effect on control animals (PIC-Sur v Sal-Sur; Figs. 8 and 9D). Since nAchRα7 expression was not diminished in sham-treated MIA animals, we concluded that a structural decrease in is not a core feature of pathogenesis in this model. However, since expression was increased nearly 100% by antipurinergic therapy, it appears that increased cholinergic signaling through the nAchRα7 receptor may be therapeutic in the MIA model of autism spectrum disorders. Cholinergic signaling through these receptors is a well-established antiinflammatory regulator of innate immunity in both the CNS [48] and periphery [59], and is dysregulated in human autism [60]. Antipurinergic therapy appears to provide a novel means for upregulating the expression of this receptor pharmacologically in disorders associated with innate immune dysregulation and inflammation.

## Conclusions

Antipurinergic therapy with suramin corrected all of the core behavioral abnormalities and multisystem comorbidities that we observed in the MIA mouse model of autism spectrum disorders. The weight of the evidence from our study supports the notion that the efficacy of suramin springs from its antipurinergic properties, but additional studies will be required to prove this point. This study did not test the generality of purinergic signaling abnormalities in other animal models or in human ASD. Although our results are encouraging, we urge caution before extending our results to humans. Long-term therapy with suramin in children with autism is not an FDA-approved usage, and is not recommended because of potentially toxic side effects that can occur with prolonged treatment [61]. However, antipurinergic therapy in general offers a fresh new direction for research into the pathogenesis, and new drug development for the treatment of human autism and related spectrum disorders.

See *Supporting Information* for additional Tables and Figures.

## Supporting Information

Figure S1 **Plasma immunoglobulin to total protein ratios** were increased by suramin treatment in males (Sal-Sal = 0.34+/−0.016; Sal-Sur = 0.40+/−0.01; PIC-Sal = 0.31+/−0.008; PIC-Sur = 0.38+/−0.01; one-way ANOVA F(2,16) = 21.9; p<0.001 Newman-Keuls post hoc test; n = 3–10 males per group). Values are expressed as mean +/− SEM.
(TIF)

Figure S2 **Plasma Corticosterone.** (A) Basal plasma corticosterone levels were higher in females than males (Sal-Sal_Males_ = 73+/−23 ng/ml; Sal-Sal_Females_ = 245+/−29 ng/ml; two-way ANOVA F(1,1,1,34) = 40.21; n = 7–12 males or females per group; p<0.001). (B) Corticosterone was unchanged in females by either poly(IC) exposure or suramin treatment (two-way ANOVA F(1,37) = 0.11 (interaction), 0.16 (suramin treatment), and 0.48 (poly(IC) exposure); p = 0.74; n = 7–11 females per group). Values are expressed as mean +/− SEM.
(TIF)

Figure S3 **Comparison of Social Preference Methods.** (A) Hand-Scored Social Preference was measured by a blinded human observer. Hand-scoring was more specific than machine (Ethovision 3) scoring because actual social interactions of nose-to-nose and nose-to-tail encounters can be distinguished from non-social, center-of-mass proximity to both stranger mouse and inanimate cup. Results are in time spent with stranger mouse vs. inanimate cup from 0–5 minutes. Analyzed by 2-Way ANOVA with Bonferroni pair-wise post testing (*p<0.05; ***p<0.001; ****p<0.0001). Treatment with suramin had little effect on normal behavior (Sal-Sal vs Sal-Sur), but a strong effect in improving social behavior in the MIA group (PIC-Sal vs. PIC-Sur). Zone x treatment interaction F(3,43) = 3.72; p<0.05; n = 9–15 males per group; age = 10-weeks. (B) Ethovision-Scored Zone Time. These results are in general agreement with the hand-scored results. However, the apparent variations are greater, limiting the statistical power of the machine-scored results. Zone x treatment interaction F(3,43) = 1.96; p = 0.13; N = 9–15 males per group; age = 10 weeks.
(TIF)

Figure S4 **Females in the Poly(IC) MIA Model Showed Fewer and Milder Behavioral Symptoms than Males.** (A) Social Preference. Females were less social and more variable in their behavior than age-matched males. The greater behavioral variability decreased statistical power in females, although the trends were similar to males. N = 9–16 males and 9–12 females per group; age = 10 weeks. (B) Rotarod Latency to Fall was decreased in Poly(IC) Males. N = 9–16 males per group; age = 11 weeks. (C) Rotarod Latency to Fall was Unchanged in Poly(IC) Females. N = 9–12 females per group; age = 11 weeks. Analysis was by 1-way ANOVA with Tukey post testing.
(TIF)

Table S1 **Cohort 1 Basal Body Temperature at 16 weeks was Decreased in the MIA Model and Restored to Normal by Antipurinergic Therapy.**
(TIF)

Table S2 **Cohort 2 Basal Body Temperature from 8 to 16 weeks was Decreased in the MIA Model and Restored to Normal by Antipurinergic Therapy.**
(TIF)

Table S3 **Circadian Analysis of Basal Metabolic Rates, Motor Activity, and Feeding.**
(TIF)
