# Supplementary figures and images for: Antipurinergic Therapy Corrects the Autism-Like Features in the Poly(IC) Mouse Model
Source: PLoS One. 2013 Mar 13;8(3):e57380. doi: 10.1371/journal.pone.0057380 (PMC3596371; doi:10.1371/journal.pone.0057380)

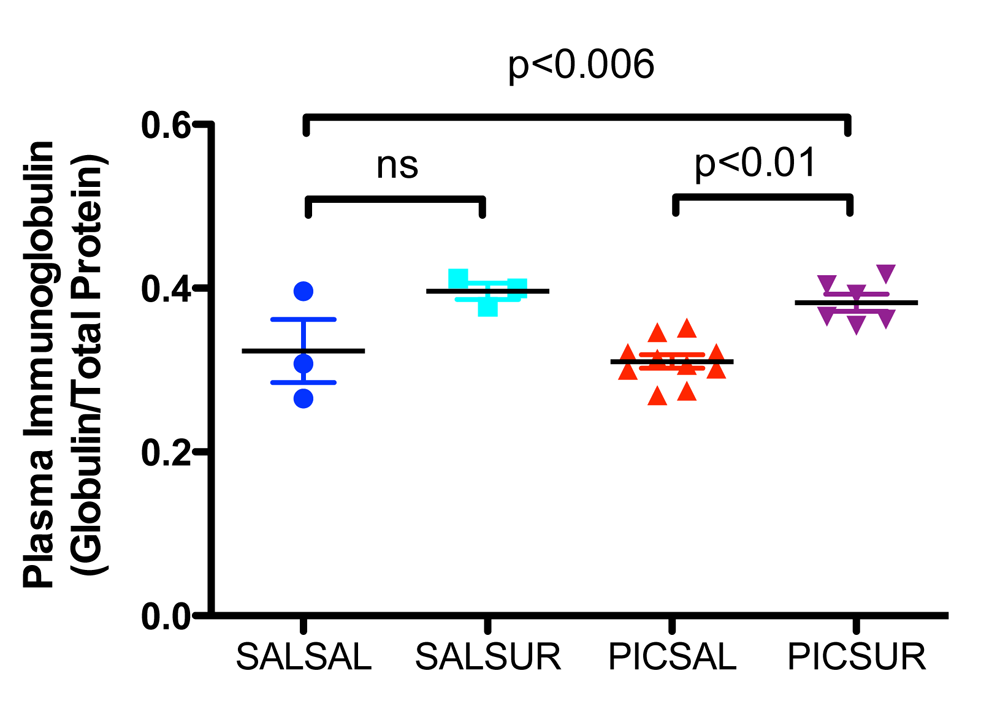

Supplement: Figure S1 — Plasma immunoglobulin to total protein ratios were increased by suramin treatment in males (Sal-Sal = 0.34+/−0.016; Sal-Sur = 0.40+/−0.01; PIC-Sal = 0.31+/−0.008; PIC-Sur = 0.38+/−0.01; one-way ANOVA F(2,16) = 21.9; p<0.001 Newman-Keuls post hoc test; n = 3–10 males per group). Values are expressed as mean +/− SEM. (TIF) [file pone.0057380.s001.tif]

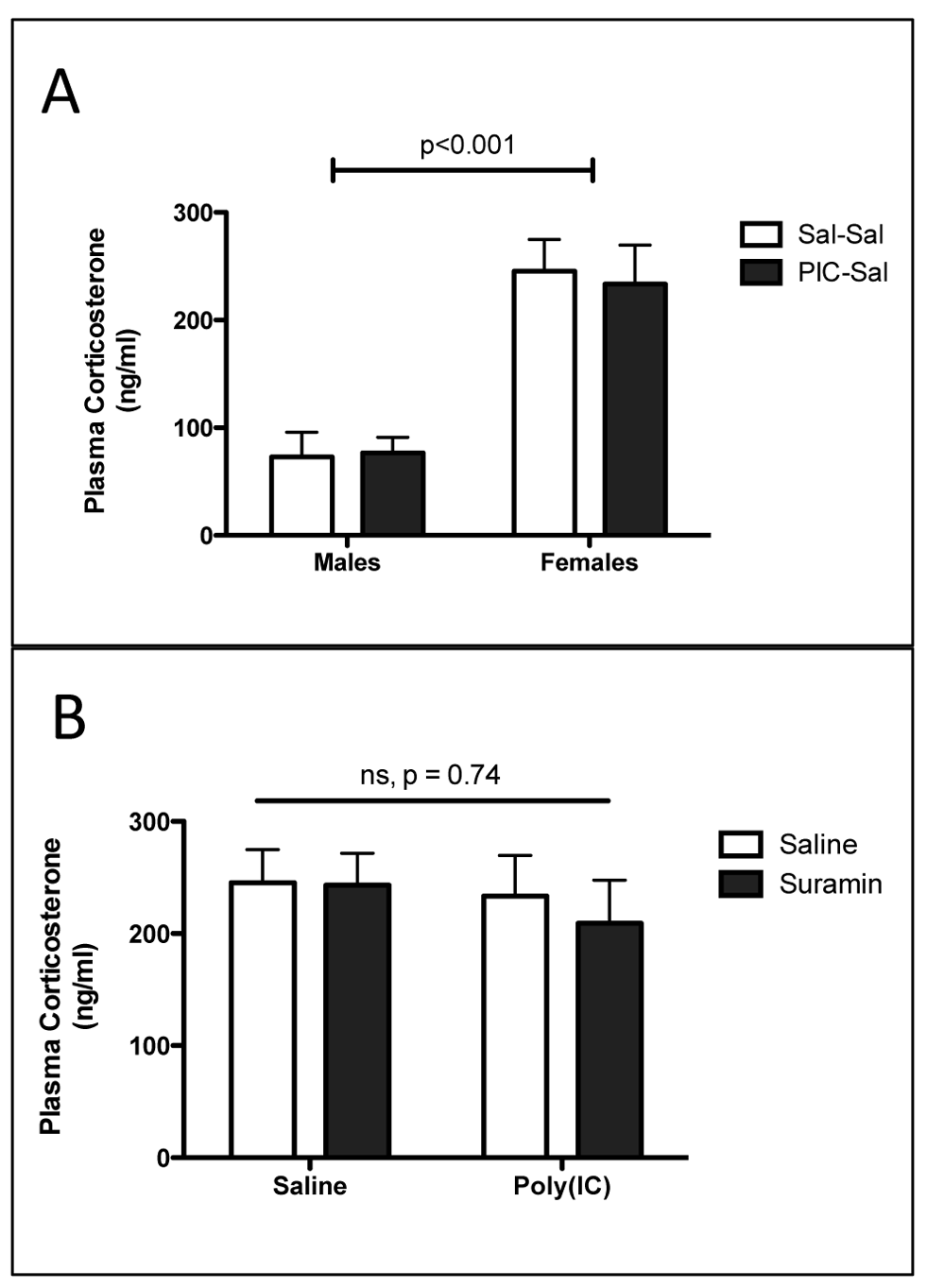

Supplement: Figure S2 — Plasma Corticosterone. (A) Basal plasma corticosterone levels were higher in females than males (Sal-SalMales = 73+/−23 ng/ml; Sal-SalFemales = 245+/−29 ng/ml; two-way ANOVA F(1,1,1,34) = 40.21; n = 7–12 males or females per group; p<0.001). (B) Corticosterone was unchanged in females by either poly(IC) exposure or suramin treatment (two-way ANOVA F(1,37) = 0.11 (interaction), 0.16 (suramin treatment), and 0.48 (poly(IC) exposure); p = 0.74; n = 7–11 females per group). Values are expressed as mean +/− SEM. (TIF) [file pone.0057380.s002.tif]

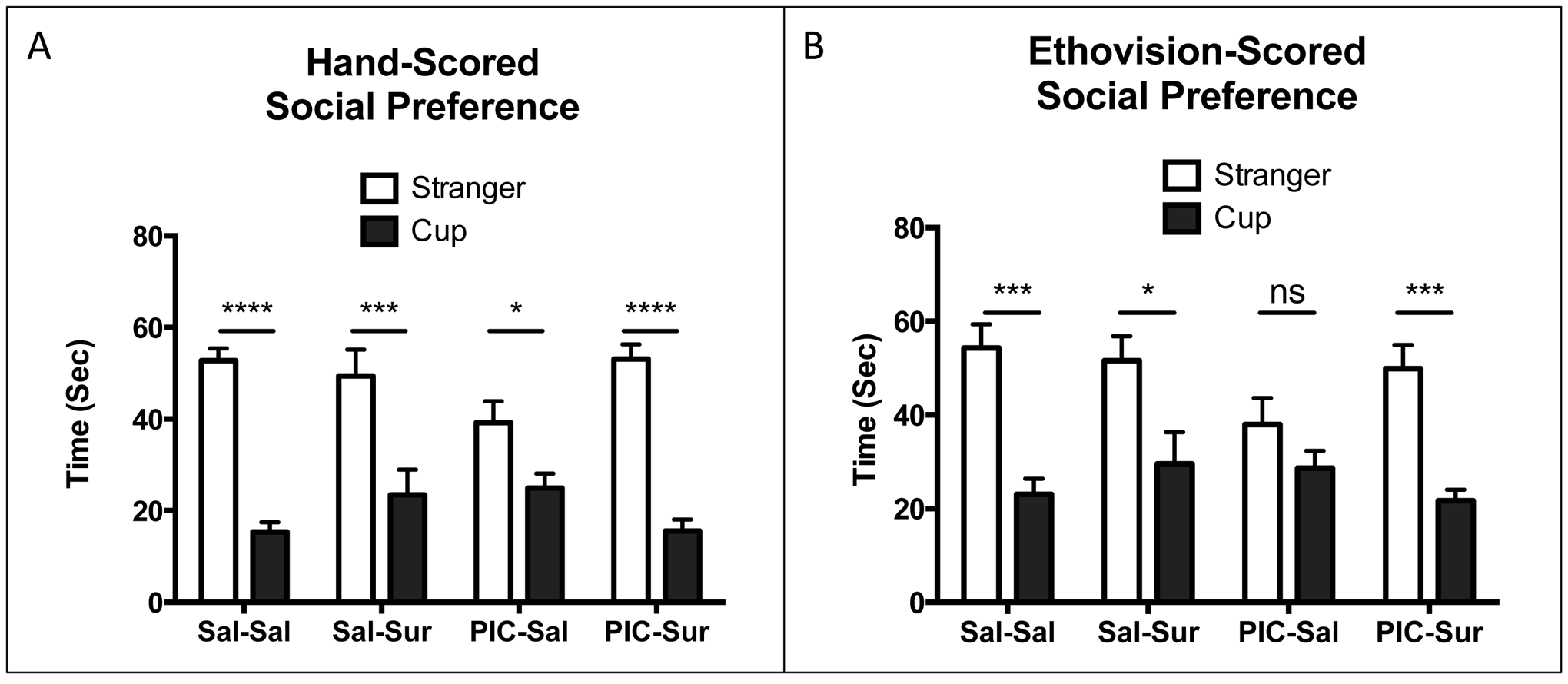

Supplement: Figure S3 — Comparison of Social Preference Methods. (A) Hand-Scored Social Preference was measured by a blinded human observer. Hand-scoring was more specific than machine (Ethovision 3) scoring because actual social interactions of nose-to-nose and nose-to-tail encounters can be distinguished from non-social, center-of-mass proximity to both stranger mouse and inanimate cup. Results are in time spent with stranger mouse vs. inanimate cup from 0–5 minutes. Analyzed by 2-Way ANOVA with Bonferroni pair-wise post testing (*p<0.05; ***p<0.001; ****p<0.0001). Treatment with suramin had little effect on normal behavior (Sal-Sal vs Sal-Sur), but a strong effect in improving social behavior in the MIA group (PIC-Sal vs. PIC-Sur). Zone x treatment interaction F(3,43) = 3.72; p<0.05; n = 9–15 males per group; age = 10-weeks. (B) Ethovision-Scored Zone Time. These results are in general agreement with the hand-scored results. However, the apparent variations are greater, limiting the statistical power of the machine-scored results. Zone x treatment interaction F(3,43) = 1.96; p = 0.13; N = 9–15 males per group; age = 10 weeks. (TIF) [file pone.0057380.s003.tif]

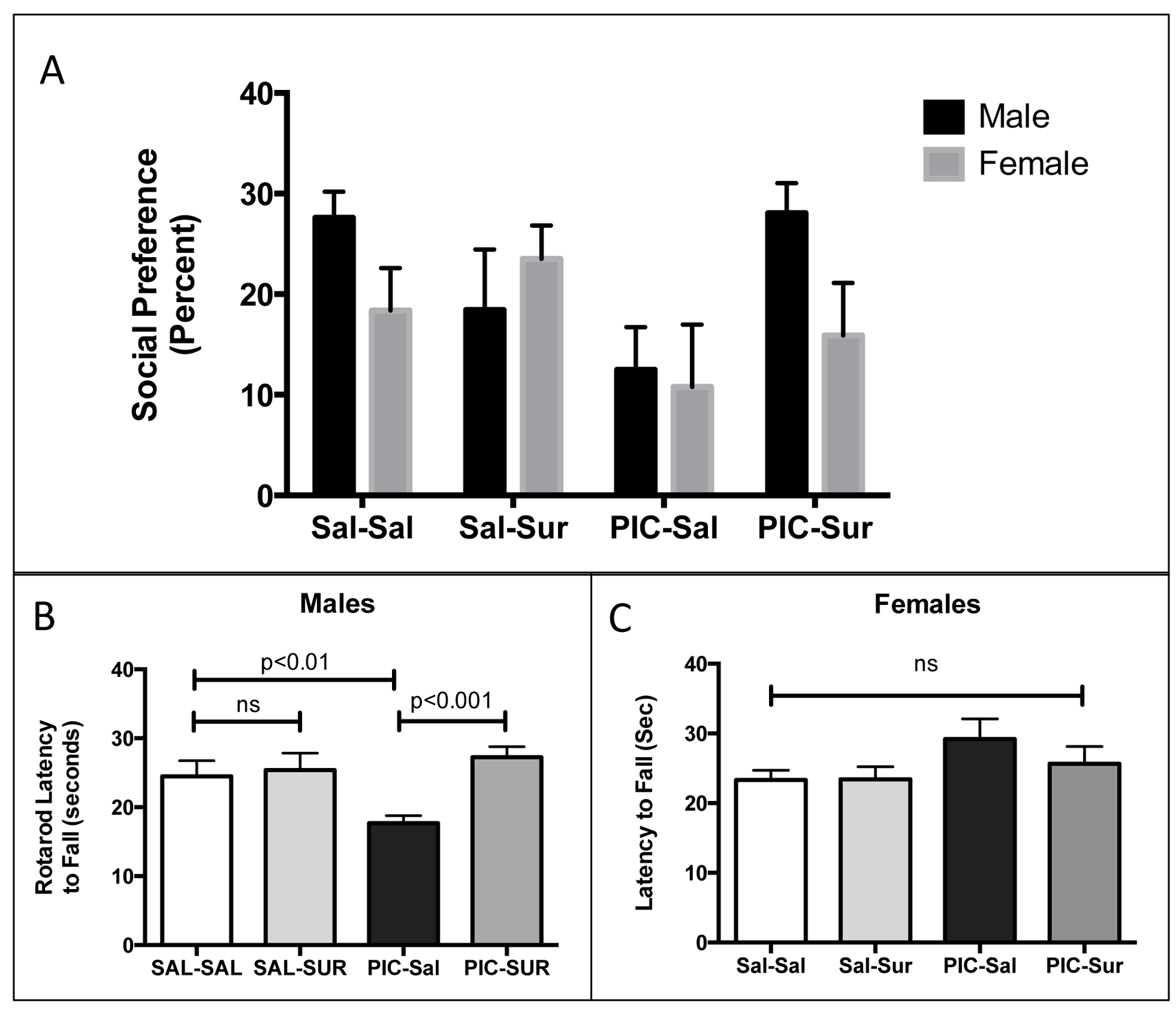

Supplement: Figure S4 — Females in the Poly(IC) MIA Model Showed Fewer and Milder Behavioral Symptoms than Males. (A) Social Preference. Females were less social and more variable in their behavior than age-matched males. The greater behavioral variability decreased statistical power in females, although the trends were similar to males. N = 9–16 males and 9–12 females per group; age = 10 weeks. (B) Rotarod Latency to Fall was decreased in Poly(IC) Males. N = 9–16 males per group; age = 11 weeks. (C) Rotarod Latency to Fall was Unchanged in Poly(IC) Females. N = 9–12 females per group; age = 11 weeks. Analysis was by 1-way ANOVA with Tukey post testing. (TIF) [file pone.0057380.s004.tif]

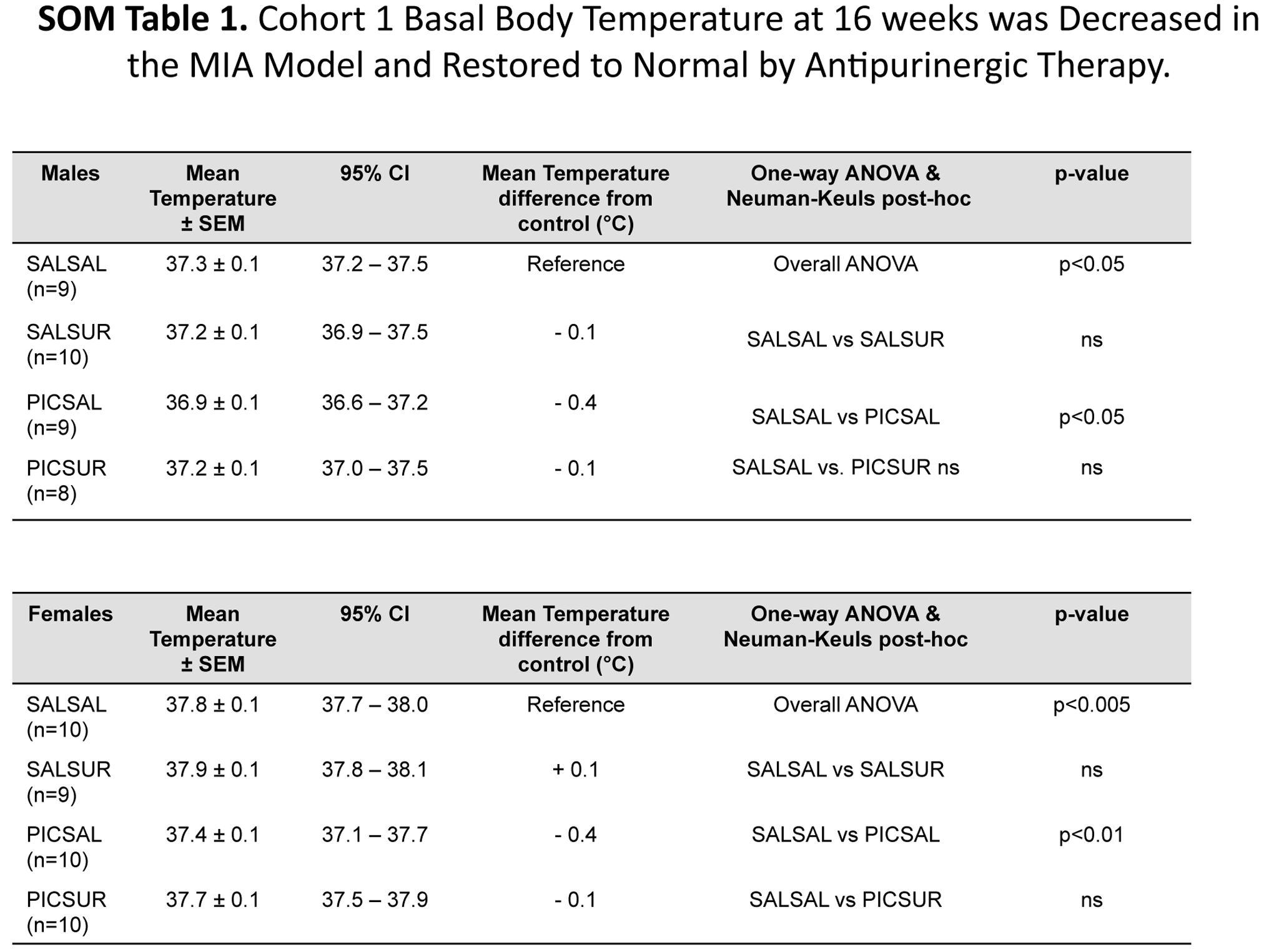

Supplement: Table S1 — Cohort 1 Basal Body Temperature at 16 weeks was Decreased in the MIA Model and Restored to Normal by Antipurinergic Therapy. (TIF) [file pone.0057380.s005.tif]

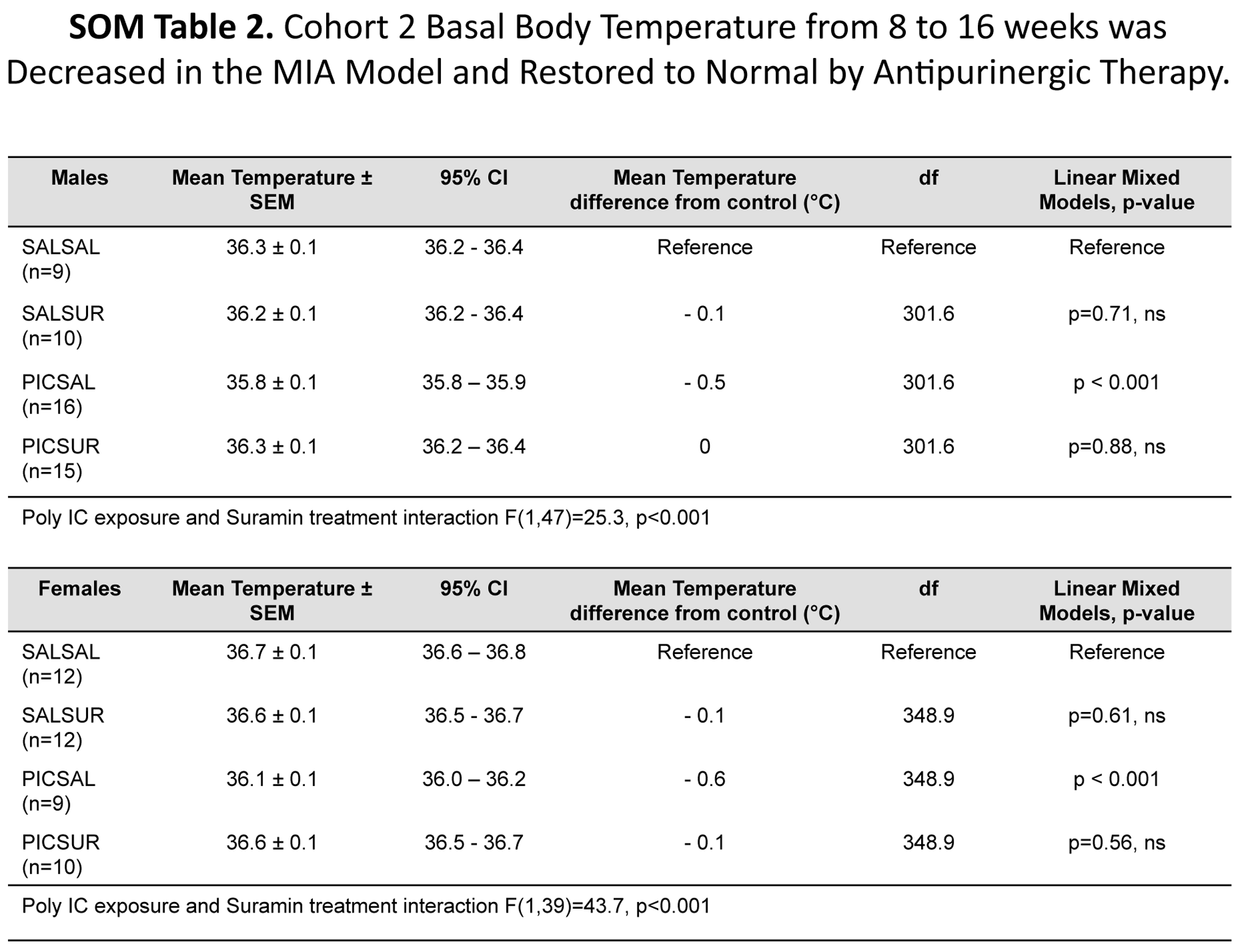

Supplement: Table S2 — Cohort 2 Basal Body Temperature from 8 to 16 weeks was Decreased in the MIA Model and Restored to Normal by Antipurinergic Therapy. (TIF) [file pone.0057380.s006.tif]

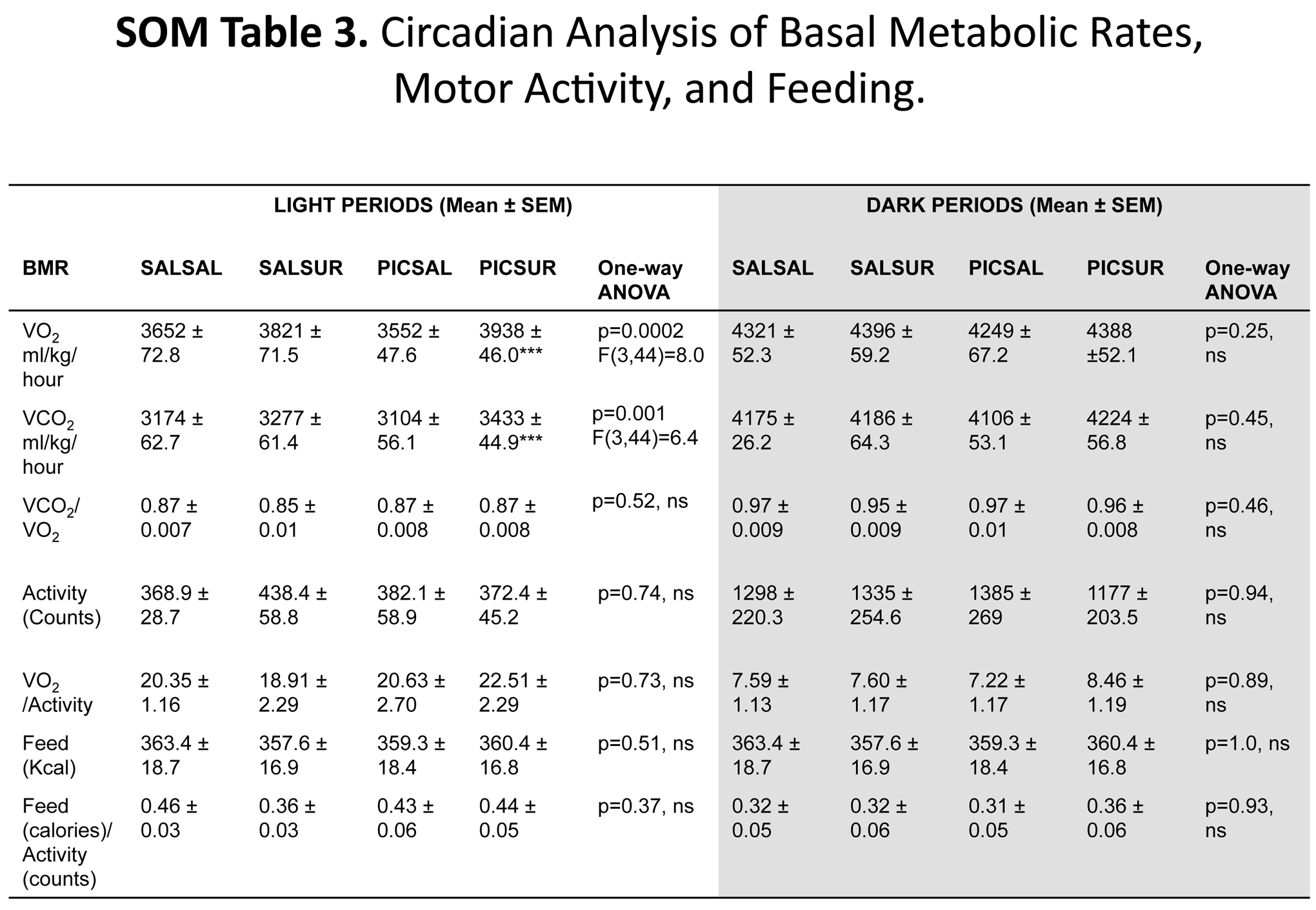

Supplement: Table S3 — Circadian Analysis of Basal Metabolic Rates, Motor Activity, and Feeding. (TIF) [file pone.0057380.s007.tif]
